# Gut microbiota composition reflects disease progression, severity and outcome, and dysfunctional immune responses in patients with hypertensive intracerebral hemorrhage

**DOI:** 10.3389/fimmu.2022.869846

**Published:** 2022-10-14

**Authors:** Jielian Luo, Yang Chen, Guanghai Tang, Zhuo Li, Xiaobo Yang, Xiaoxiao Shang, Tao Huang, Gan Huang, Lixin Wang, Yun Han, Yuexiang Zhou, Chuyang Wang, Bin Wu, Qihua Guo, Baoying Gong, Mengzhen Li, Ruihua Wang, Jiecong Yang, Wanzhen Cui, Jianbin Zhong, Linda Ld Zhong, Jianwen Guo

**Affiliations:** ^1^ The Second Clinical Medical College, Guangzhou University of Chinese Medicine, Guangzhou, China; ^2^ Guangdong Provincial Hospital of Chinese Medicine, Guangzhou, China; ^3^ State Key Laboratory of Dampness Syndrome of Chinese Medicine, The Second Affiliated Hospital of Guangzhou University of Chinese Medicine, Guangzhou, China; ^4^ Department of Neurology, Shenyang Second Hospital of Traditional Chinese Medicine, Shenyang, China; ^5^ Genetic Testing Lab, The Second Affiliated Hospital of Guangzhou University of Chinese Medicine, Guangzhou, China; ^6^ Guangdong Provincial Key Laboratory of Clinical Research on Traditional Chinese Medicine Syndrome, Guangzhou, China; ^7^ Department of Neurology, The Second Affiliated Hospital of Guangzhou University of Chinese Medicine, Guangzhou, China; ^8^ Department of Neurology, Yangjiang Hospital of Traditional Chinese Medicine, Yangjiang, China; ^9^ Department of Intensive Care Unit, The Second Affiliated Hospital of Guangzhou University of Chinese Medicine, Guangzhou, China; ^10^ Department of Community Healthcare Service, Shenzhen FuYong People’s Hospital, Shenzhen, China; ^11^ Biological Resource Center, The Second Affiliated Hospital of Guangzhou University of Chinese Medicine, Guangzhou, China; ^12^ The Fourth Affiliated Hospital of Guangzhou Medical University Research Team of Traditional Chinese Medicine for the Prevention and Treatment of Cerebral Hemorrhage, The Second Affiliated Hospital of Guangzhou University of Chinese Medicine, Guangzhou, China; ^13^ Department of Neurology, The Fourth Affiliated Hospital of Guangzhou Medical University, Guangzhou, China; ^14^ Hong Kong Chinese Medicine Clinical Study Centre, School of Chinese Medicine, Hong Kong Baptist University, Hong Kong, Hong Kong SAR, China

**Keywords:** intracerebral hemorrhage, stroke-associated pneumonia, gut microbiota, *Enterococcus*, *Prevotella*, cytokines

## Abstract

**Objective:**

In this study, we aimed to explore the alterations in gut microbiota composition and cytokine responses related to disease progression, severity, and outcomes in patients with hypertensive intracerebral hemorrhage (ICH).

**Methods:**

Fecal microbiota communities of 64 patients with ICH, 46 coronary heart disease controls, and 23 healthy controls were measured by sequencing the V3-V4 region of the 16S ribosomal RNA (16S rRNA) gene. Serum concentrations of a broad spectrum of cytokines were examined by liquid chips and ELISA. Relationships between clinical phenotypes, microbiotas, and cytokine responses were analyzed in the group with ICH and stroke-associated pneumonia (SAP), the major complication of ICH.

**Results:**

In comparison with the control groups, the gut microbiota of the patients with ICH had increased microbial richness and diversity, an expanded spectrum of facultative anaerobes and opportunistic pathogens, and depletion of anaerobes. *Enterococcus* enrichment and *Prevotella* depletion were more significant in the ICH group and were associated with the severity and functional outcome of ICH. Furthermore, *Enterococcus* enrichment and *Prevotella* depletion were also noted in the SAP group in contrast to the non-SAP group. *Enterococci* were also promising factors in the prognosis of ICH. The onset of ICH induced massive, rapid activation of the peripheral immune system. There were 12 cytokines (Eotaxin, GM-CSF, IL-8, IL-9, IL-10, IL-12p70, IL-15, IL-23, IL-1RA, IP-10, RANTES, and TNF-α) changed significantly with prolongation of ICH, and the Th2 responses correlated with the 90-day outcomes. Cytokines TNF-α, IP-10, IL-1RA, IL-8, IL-18, and MIP-1β in SAP group significantly differed from non-SAP group. Among these cytokines, only IP-10 levels decreased in the SAP group. *Enterococcus* was positively associated with IL-1RA and negatively associated with IP-10, while *Prevotella was* inversely associated in both the ICH and SAP groups.

**Conclusion:**

This study revealed that gut dysbiosis with enriched *Enterococcus* and depleted *Prevotella* increased the risk of ICH and subsequently SAP. The altered gut microbiota composition and serum cytokine profiles are potential biomarkers that reflect the inciting physiologic insult/stress involved with ICH.

## Introduction

Intracerebral hemorrhage (ICH) is a devastating disease and a major public health issue worldwide (1). The fatality and long-term mortality rate of ICH have not changed significantly in recent years (2), and effective treatments have not yet been found in the internal medicine and surgery fields (3–7). Thus, alternative treatment options are needed to improve ICH prognosis and lower the risk of mortality.

The gut-brain axis is a bidirectional communication system that involves multiple pathways including neural, hormonal, and immunological signals with the microbiota as the central mediator (8). Acute ischemia rapidly causes severe gastrointestinal paralysis, ischemia and produces excess nitrate leading to intestinal dysbiosis (9, 10). In turn, the intestinal flora and their metabolites affect the outcome and prognosis of stroke. The use of broad-spectrum antibiotics before a stroke can reduce the overall diversity of intestinal microbes and reduce cerebral infarction, which involves intestinal immune cell traffic to the meninges (11). Further, germ-free mice with intestinal dysbiosis after stroke had increased lesion volume and functional impairment compared to normal control mice (9). Alterations in gut microbiota composition affect the host immune system with inflammatory cytokine production and immune cell differentiation (12), enhancing the role of neuroinflammation in ICH. Thus, the intestinal microbiota and the interplay with the immune system are intervention strategies in the setting of stroke. Notably, current studies focus on ischemic stroke while the alteration of the microbiota and relative immune system in ICH are less studied, particularly *in vivo*. Here, we characterized the microbiota and peripheral cytokine alterations in ICH patients and analyzed the relationships between changes in fecal microbiota and immune responses.

## Materials and methods

**Figure 1:**
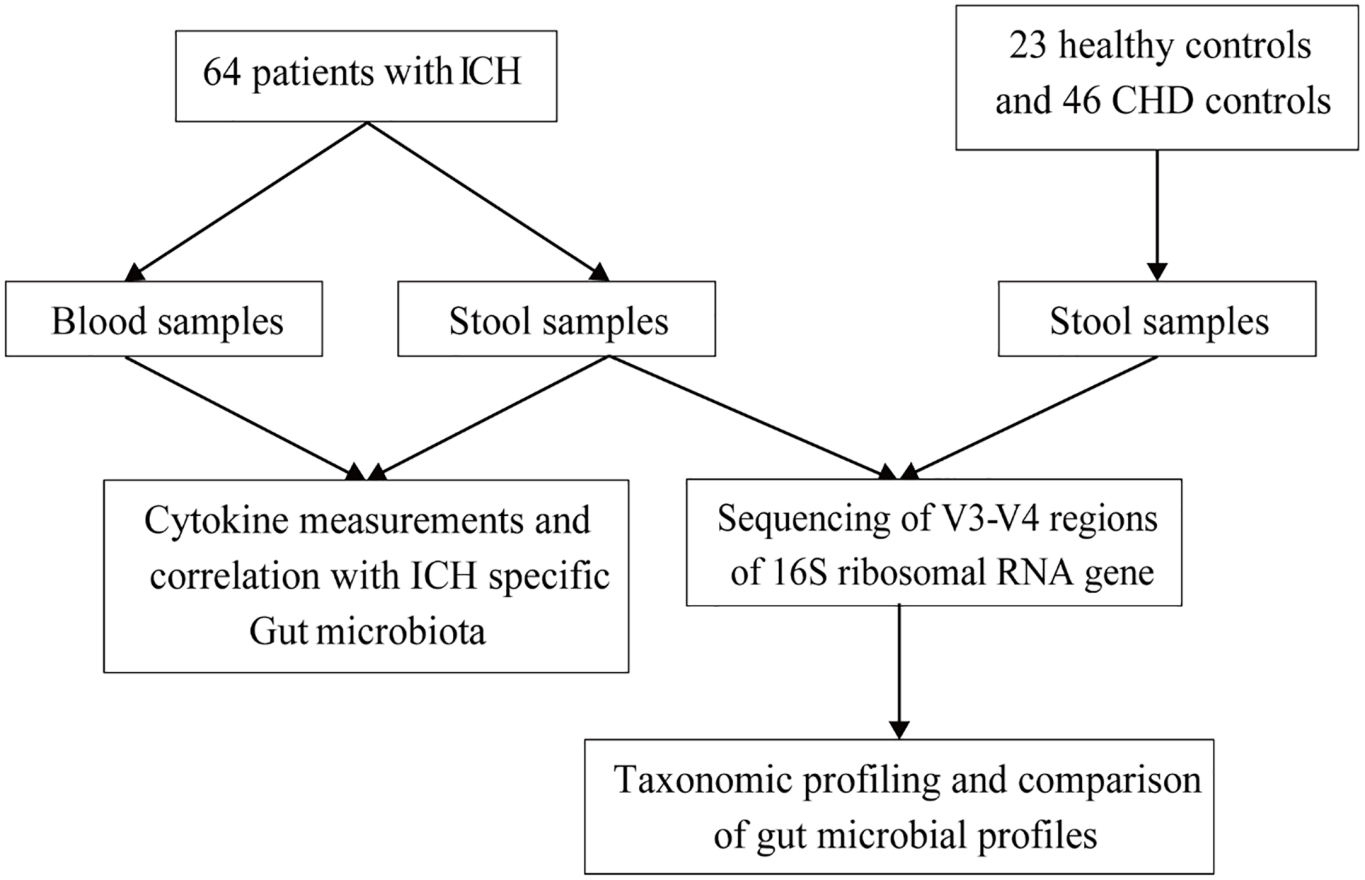
Flow chart and enrolled participants in the current study.

The flow chart is shown in **Figure 1**. A total of 64 ICH patients, 46 coronary heart disease (CHD) controls, and 23 healthy controls were enrolled from April 2018 to December 2020 in the neurology department of Guangdong Provincial Hospital of Traditional Chinese Medicine, Shenyang Second Hospital of Traditional Chinese Medicine, The Fourth Affiliated Hospital of Guangzhou Medical University, and the health examination department of Guangdong Provincial Hospital of Traditional Chinese Medicine. All subjects provided written informed consent to participate in this study.

ICH was diagnosed according to American Heart Association/American Stroke Association guidelines (13). Stroke-associated pneumonia (SAP) following ICH is the most common complication after stroke. In this study, the diagnosis of SAP was based on modified Centers for Disease Control and Prevention (CDC) criteria (14). The inclusion criteria were as follows (1) age >18 years, (2) admission within 7 days of ICH onset, and (3) informed consent obtained and the retention of biological samples completed. The exclusion criteria were as follows: (1) ICH caused by brain tumor, brain trauma, blood diseases, cerebrovascular malformation, or aneurysm, (2) any antibiotics, prebiotics, or probiotics treatment within four weeks before admission, (3) active infection within two weeks before admission, (4) liver and kidney dysfunction, (5) history of gastrointestinal diseases such as gastrointestinal tumor, inflammatory bowel disease, or active gastrointestinal bleeding in the last 3 months, and (6) history of immune-related diseases or receiving immunotherapy. Clinical data such as age, gender, medical history, and neurological deficits were assessed and collected by neurologists.

### Sample collection

Stool and serum samples of patients with ICH were collected at T1 (0–3 days after ICH), T2 (4–7 days after ICH), T3 (8–14 days after ICH), and T4 (14–30 days after ICH). In total, 170 stool and 184 serum samples were collected after the onset of symptoms. The number of stool samples for T1 to T4 were 44, 33, 82, and 11, while the number of serum samples for T1 to T4 were 46, 34, 88, and 16. Venous blood (10 mL) was collected at different phases on an empty stomach and centrifuged (3000 rpm, 10 min) within 6 h after collection. After centrifugation, the serum was divided into cryovials and stored in an -80°C refrigerator for cytokine analysis. Fecal Samples were collected in the morning, stored in the Fecal Microbial Collection and Preservation Kit (ML-001A, Shenzhen Dayun Gene Technology Co., Ltd.), and saved in an -80°C refrigerator within 72 h. Stool samples of healthy controls (HC) and CHD group were collected using the same methods. The specimens of all collaborative subcenters were transported through a cold chain and stored uniformly in the Biological Resource Center of Guangdong Provincial Hospital of Traditional Chinese Medicine to avoid repeated freezing and thawing.

### DNA extraction, 16S ribosomal RNA gene sequencing

According to the manufacturer’s instructions, the DNA was extracted using the magnetic soil and stool genomic DNA extraction kit (Magnetic Soil and Stool DNA Kit, Tiangen Biochemical Technology Co., Ltd.). After extracting total DNA from the stool samples, we used primers, 341F (CCTAYGGGRBGCASCAG) and 806R (GGACTACNNGGGTATCTAAT), to amplify the V3-V4 region of the bacterial 16S rRNA gene. The library was constructed using TruSeq DNA PCR-Free Library Preparation Kit from Illumina. The constructed library was subjected to Qubit quantification and library testing. After it was quantified, the NovaSeq 6000 was used for sequencing.

### Measurement of serum cytokine levels by liquid chips and ELISA

This study examined 36 cytokines at different time points in patients with ICH. The list of cytokines is: MIP-1α, SDF-1α, IL-27, IL-1 β, IL-2, IL-4, IL-5, IP- 10. IL-6, IL-7, IL-8, IL-10, Eotaxin, IL-12p70, IL-13, IL-17A, IL-31, IL-1RA, RANTES, IFN-γ, GM-CSF, TNF-α, MIP-1β, IFN-α, MCP-1, IL-9, TNF-β, CXCL-1, IL-1α, IL-23, IL-15, IL-18, IL-21, IL- 22, CXCL-2 and TGF-β. Among these cytokines, CXCL-2 (Shanghai Lianshuo Biotechnology, AE91213Hu) and TGF-β (Shanghai Lianshuo Biotechnology, AE98029Hu) were tested by ELISA according to the manufacturer’s instructions. The remaining cytokines were assessed by a multiplex liquid-chip assay based on Luminex xMAP. Samples with cytokine values below the lower limit of detection were designated as the lower limit of detection for that specific cytokine. The detection limit was 0.3 ~ 13.7 pg/mL.

### Statistical analyses

Categorical variables are presented as numbers and percentages, and continuous variables are presented as mean ± standard deviation (SD) or median (interquartile range (IQR)). Comparisons between groups were performed with chi-square tests for categorical variables. Continuous variables that followed the normal distribution were compared with the Student’s t test or analysis of variance (ANOVA). Variables inconsistent with the normal distribution and Levene’s test were compared with the non-parametric Wilcoxon test or Kruskal-Wallis test. Spearman’s rank correlations were calculated between the relative abundance of bacterial communities and environmental variables or cytokine responses. The predictive performance of ICH prognosis was assessed by comparing receiver operating characteristic (ROC) curves. Statistical analysis was performed using SPSS 26.0 (Statistical Package for Social Sciences, Chicago, IL, USA) software. The analysis of intestinal flora was performed using QIIME software (version 1.9.1) and the R language tool (version 3.4.0). Changes in cytokine concentration were plotted using GraphPad Prism 9 (GraphPad Software, Inc.) software. A two-sided, P<0.05 was considered statistically significant.

## Results

### Clinical characteristics of ICH and control groups

The demographic and clinical information of the 64 ICH patients, 46 CHD controls, and 23 healthy subjects included in this study are shown in **Table S1** in the appendix. As **Table S1** shows, there was no statistical difference in age and gender between the ICH group and the CHD group. Compared with the CHD group, the ICH group had patients with a higher proportion of hypertension history (76.563% *vs* 26.087%, P < 0.001). The two groups had similar rates of smoking and alcohol abuse histories. The median triglyceride in the CHD group was higher than that in the ICH group (0.995 *vs* 1.43, P=0.012), while the levels of total cholesterol, low-density lipoprotein, and high-density lipoprotein were similar in the two groups. Compared with the HC group, the proportion of men in the ICH group was higher (59.375% *vs* 26.087%, P = 0.006) and the average age was older (P < 0.001). The proportion of patients with a history of hypertension, active smoking, drinking, and coronary heart disease was higher in the ICH group than in the HC group. Additionally, the median (interquartile range) of the ICH scores, GCS at admission, and NIHSS score at admission of the ICH group were 1 (2), 14 (6), and 10 (9) respectively. There were 27 (42.20%) patients with a neurological recovery defined as NIHSS score improving ≥ 40% after 14 d of standard treatments. In addition, there were 37 patients (57.81%) with functional independence (mRS ≤ 2) at 90 d.

### Patients with ICH have altered and more diverse gut microbiota

**Figure 2:**
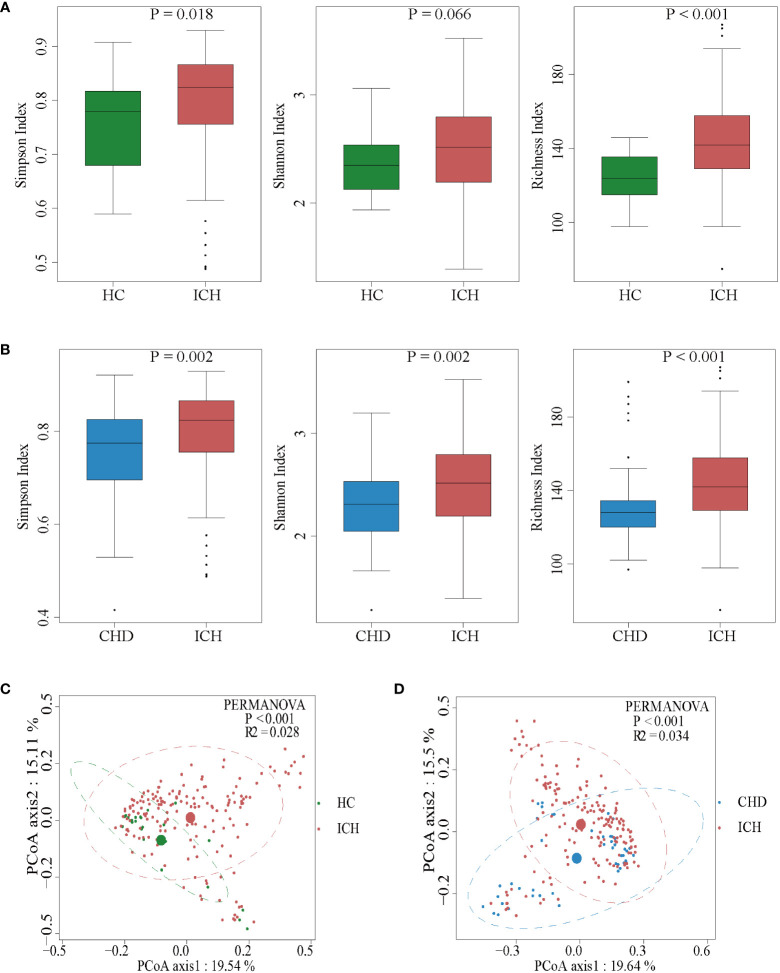
Comparison of the microbial communities of the ICH and control groups. **(A, B)** Box plots depict differences in the fecal microbiota diversity indices between the ICH and control groups according to the Simpson index, Shannon index, and richness index based on OTU counts. Each box plot represents the median, interquartile range, minimum, and maximum values. OUT: operational taxonomic units. **(C, D)** PCoA with the Bray-Curtis dissimilarities showing the gut microbiota composition among healthy controls **(C)** or CHD controls **(D)** and the patients with ICH.

We then characterized the ICH-associated gut microbiota by high-throughput sequencing of the V3-V4 region of the 16S rRNA gene. The gut microbial composition is shown in **Figure S1A**. The bacterial diversity and richness in the ICH and the control groups were measured by different methods using the Simpson index, Shannon index, and richness index. ICH patients had more diverse gut microbiota than the controls (Wilcoxon rank-sum test, compared to HC group, P = 0.018 for the Simpson index and P < 0.001 for the richness index, **Figure 2A**; compared to CHD group, P = 0.002 for the Simpson index and Shannon index and P < 0.001 for the richness index, **Figure 2B**). Specifically, these results remained significant according to longitudinal analyses (Kruskal-Wallis Test, compared to HC group, P = 0.036 for the Shannon index, P < 0.001 for the richness index, **Figure S1B**; compared to CHD group, P = 0.027 for the Simpson index, P = 0.009 for the Shannon index and P < 0.001 for the richness index, **Figure S1C**).

To determine whether there were significant differences in the microbiota structure between ICH patients and controls, principal coordinate analysis (PCoA) was used. The microbial composition of the ICH group was significantly different from that of the HC or CHD group according to Bray-Curtis differences (Permutational multivariate analysis of variance (PERMANOVA) test; ICH *vs* HC, R2 = 0.028, P < 0.001; ICH *vs* CHD, R2 = 0.034, P < 0.001**)** (**Figures 2C, D**). Furthermore, the PCoA also revealed that the gut microbiota changed dynamically with the prolongation of ICH (**Figures S1D, E**). These results suggested that the richness and diversity of gut microbiota in patients with ICH were significantly different from those of controls.

### The gut microbiota profile shows *Enterococcus* enrichment and *Prevotella* depletion in the ICH group

**Figure 3:**
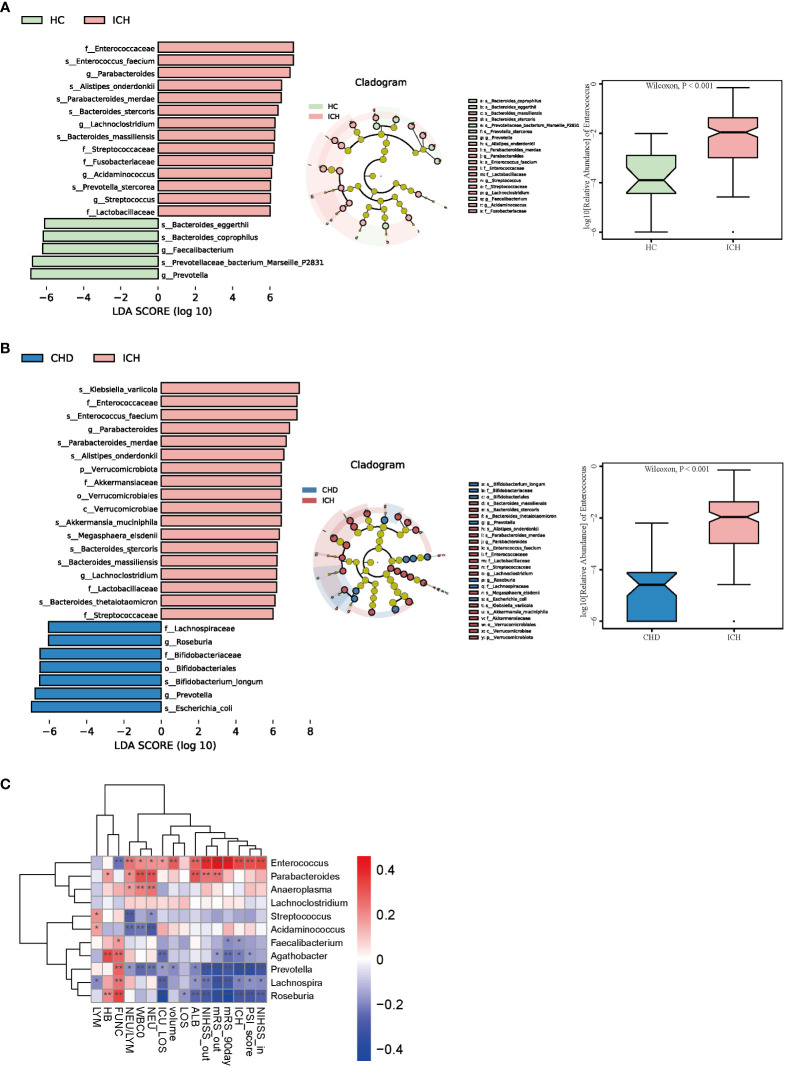
Taxonomic differences of fecal microbiota in the ICH and control groups. **(A, B)** LEfSe analysis revealed significant bacterial differences in fecal microbiota between the ICH (positive score) and control groups (negative score). LDA scores (log10) > 6 and P < 0.05 are shown (left panel). Cladogram using the LEfSe method indicating the phylogenetic distribution of fecal microbiota associated with the ICH and control participants (median panel). The relative abundance of *Enterococcus* was significantly higher in the ICH group than in the control groups. (P < 0.001) (right panel). **(C)** Heatmap of Spearman’s rank correlation coefficient among clinical indexes and 11 genera (LDA scores (log10) > 4). *: P < 0.05, **: P < 0.01.

To identify the most relevant taxa responsible for the observed differences, supervised comparisons of the microbiota between the ICH and control groups were performed by linear discriminant analysis (LDA) effect size (LEfSe) analysis without any adjustment. We used a logarithmic LDA score cutoff of 6.0 to identify important taxonomic differences between the ICH and control groups and found a notable difference in fecal microbiota. We identified, through LEfSe analysis, 19 taxa that were differentially abundant in the HC and ICH groups (**Figure 3A**) and 25 taxa in the CHD and ICH groups (**Figure 3B**). We observed that the relative abundances of *Prevotella* and *Faecalibacterium* were higher in the HC group than those in the ICH group, while the relative abundances of *Enterococcus*, *Parabacteroides*, *Lachnoclostridium*, *Acidaminococcus*, and *Streptococcus* were higher in the ICH group than those in the HC group. Moreover, the relative abundances of *Prevotella* and *Roseburia* were higher in the CHD group, whereas the relative abundances of *Enterococcus*, *Parabacteroides*, and *Lachnoclostridium* were higher in the ICH group. Notably, the relative abundance of *Enterococcus* was higher in patients with ICH compared to controls (**Figures 3A, B**). Additionally, significant taxa were observed at different times after ICH (**Figure S2A, B**). A generalized linear model (GLM) was used to model the microbiota that were significantly different between the ICH and control groups after controlling for possible confounding factors (age, gender, antibiotic use, and comorbidities) (15). As **Tables S2A, B** shows, *Enterococcus*, *Parabacteroides*, *Streptococcus*, *Veillonella*, *Clostridium _innocuum_group*, and *Eubacterium_eligens_group* differed significantly between the ICH and control groups after adjustment. Further, we found that the ICH score was the most important phenotype that contributed to the flora variation in ICH (**Figure S2D**), suggesting that ICH was the major cause of microbiota alteration rather than hypertension or other comorbidities.

In addition, we found that the relative abundance of *Enterococcus* increased with a prolonged duration of ICH and was highest in the T4 phase **(** **Figure S2C**). *Enterococcus* was correlated positively with the severity of ICH (admission and discharge NIHSS (r = 0.337 and r = 0.394, P < 0.001), PSI score (r = 0.324, P < 0.001), ICH score(r = 0.339, P < 0.001), hematoma volume(r = 0.289, P < 0.001), length of ICU stay (r = 0.181, P = 0.025), neutrophil-to-lymphocyte ratio (NLR) (r = 0.246, P = 0.002), and poor outcome of ICH (discharge and 90-day mRS scores(r = 0.462 and r= 0.432, P < 0.001)) (**Figure 3C**). Conversely, *Prevotella* and *Roseburia* were negatively related to the severity and poor outcomes of ICH (**Figure 3C**).

To understand microbial community metabolism among the ICH and control groups, MetaCyc was used, which is a database of metabolic pathways and components covering all domains of life (16). It showed that peptidoglycan biosynthesis V (β-lactam resistance) (PWY-6470), the super pathway of β-D-glucuronosides degradation (GLUCUROCAT-PWY), Bifidobacterium shunt (P124-PWY), and hexitol fermentation to lactate, formate, ethanol, and acetate(P461-PWY) were enriched in the ICH group (**Figure S2E**), which were also positively correlated with Enterococcus (r = 0.866, r = 0.659, r = 0.697 and r = 0.655, respectively, P <0.001) (**Figure S2F**). The functional capacities of the intestinal microbiome were predicted based on 16S data using BugBase (17). At the organism level, three potential phenotypes including anaerobic, facultatively anaerobic, and containing mobile elements were predicted to be significant in the ICH and control groups (P < 0.001) as **Figure S3** shows. Among these three phenotypes, the ICH group had more mobile elements and facultative anaerobic bacteria and less anaerobic bacteria as the disease progressed. Meanwhile, the proportion of the facultative anaerobia phenotype was significantly enriched in the ICH group in the T1-T3 phases, with a mild recovery in phase T4. Collectively, these results suggested changes in microbiota profile were closely related to the disrupted intestinal microenvironment.

### *Enterococcus* enrichment and *Prevotella* depletion in the SAP group

We next examined the association between fecal microbiota and ICH complications. Among the 64 ICH patients, 47 (73.4%) were initially diagnosed with pneumonia (SAP), while the remaining patients did not have SAP (non-SAP). The demographic and clinical information of the 47 patients with SAP and 17 non-SAP subjects in the T1 phase are shown in **Table S3**. The SAP group had similar age and gender as the non-SAP group. Additionally, the SAP group had higher rates of midline shift (51.064% versus 11.765%, P = 0.008) and was likely to have larger hematoma volume (median (IQR), 13 (21) versus 6.54 (12.5), P = 0.007), higher initial white blood cell count (median (IQR), 10.7 (4.65) versus 6.92 (4.1), P = 0.003), higher neutrophil count (median (IQR), 8.36 (4.86) versus 4.45 (4.88), P = 0.012), and higher NLR (median (IQR), 6.846 (6.112) versus 3.5 (5.406), P = 0.021). Compared to non-SAP group, the SAP group also had a higher ICH score (median (IQR), 1 (2) versus 0 (1), P < 0.001), higher GCS, and higher NIHSS on admission (median (IQR), 13 (7) versus 15 (0); 13 (12) versus 7 (5), P < 0.001). The non-SAP group had more patients with neurological recovery after 14 d of standard treatments (76.471% versus 29.787%, P = 0.001). However, there was no difference in 90-day functional independence between the two groups.

**Figure 4:**
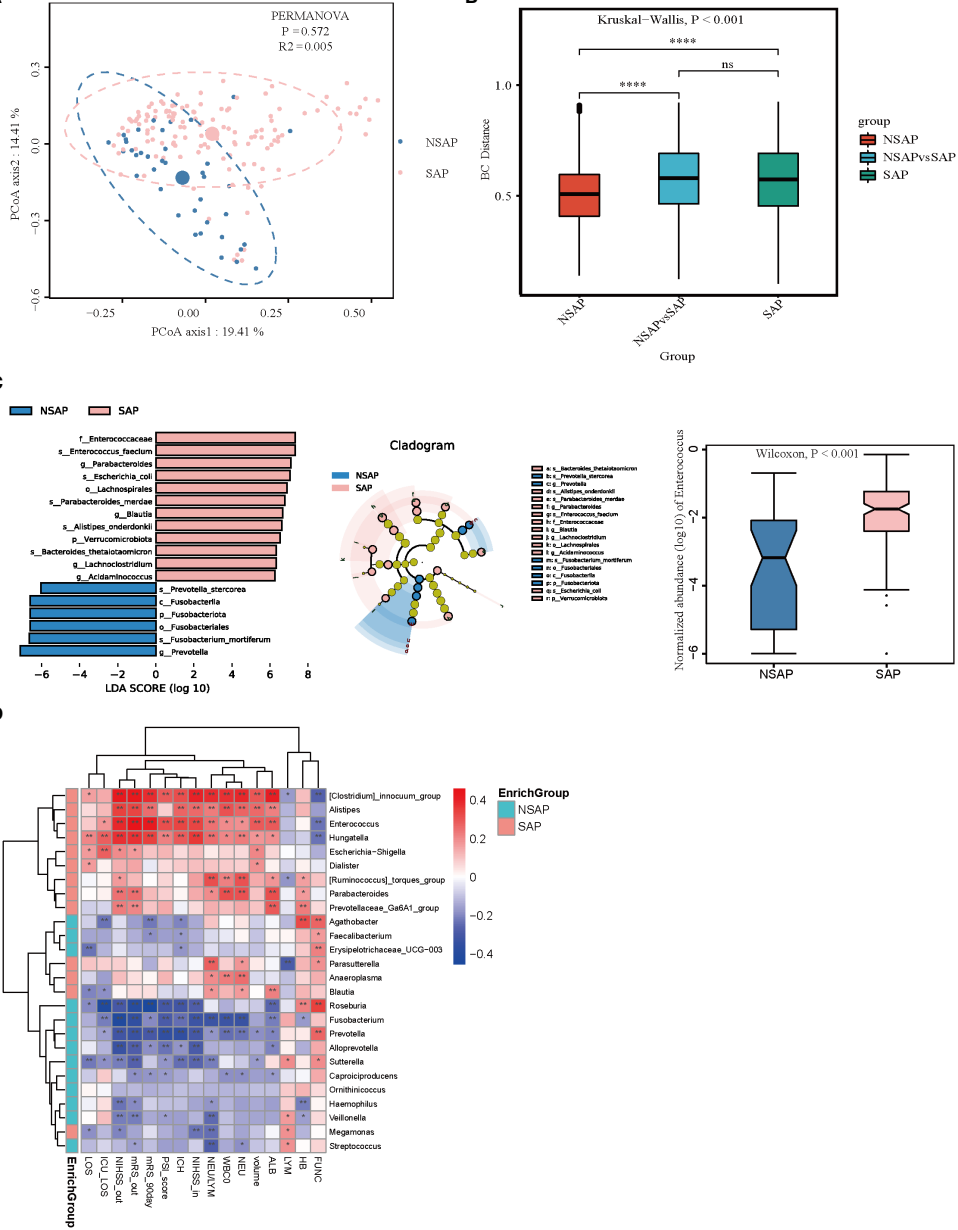
Comparison of the microbial communities of the SAP and non-SAP group. **(A)** PCoA plot with the Bray-Curtis dissimilarities demonstrates that the bacterial communities were similar between the SAP (n = 131) and non-SAP group (n = 39). **(B)** Analysis of similarities (ANOSIM) in Bray-Curtis distances showed that the SAP group differed from the non-SAP group. **(C)** LEfSe analysis revealed significant bacterial differences in fecal microbiota between the SAP (positive score) and non-SAP groups (negative score). LDA scores (log10) > 6 and P < 0.05 are shown (left panel). Cladogram using the LEfSe method indicating the phylogenetic distribution of fecal microbiota associated with the SAP and non-SAP groups (median panel). The relative abundance of *Enterococcus* was significantly higher in the SAP group than in the non-SAP group (P < 0.001) (right panel). **(D)** Heatmap of Spearman’s rank correlation coefficient among clinical indexes and significant genera (LDA scores (log10) > 4). *: P < 0.05, **: P < 0.01.

The gut microbial composition of SAP is shown in **Figure S4A**. In the PCoA analysis, there was no difference between the patients with and without SAP with respect to gut microbiota (PERMANOVA test, R2 = 0.005, P = 0.572) (**Figure 4A**). While the SAP group was different from the non-SAP group using analysis of similarities (ANOSIM) (Kruskal-Wallis test, P < 0.001) (**Figure 4B**). The dysbiosis of gut microbiota in patients with and without SAP are shown in **Figure S4B**. Furthermore, the LEfSe algorithm was used to analyze the flora with significant differences between the two groups. We found that 18 taxa were differentially abundant in the two groups (**Figure 4C**). Among them, *Enterococcus*, *Parabacteroides*, *Blautia*, *Lachnoclostridium*, and *Acidaminococcus* were significantly enriched, and *Prevotella* were depleted in patients with SAP compared to the non-SAP group. The relative abundance of *Enterococcus* was higher in patients with SAP than in non-SAP (P < 0.001) (**Figure 4C**); moreover, GLM further confirmed that the *Prevotella*, *Blautia*, *Ruminococcus_torques_group*, *Sutterella*, and *Veillonella* were different between the two groups after controlling for age, hematoma volume, NIHSS score, and antibiotic use (**Table S2C**). *Enterococcus*, *Alistipes*, *Hungatella*, and *clostridium_immocuum_group* were enriched in the SAP group and were positively correlated with the severity of ICH (admission and discharge NIHSS, ICH score, and hematoma volume), severity of pneumonia (NLR and PSI score (except *Alistipes*)), and poor outcome of ICH (discharge and 90-day mRS scores (**Figure 4D**). *Roseburia*, *Fusobacterium*, and *Prevotella* were enriched in the non-SAP group and were negatively correlated with the severity and poor outcomes of ICH. Enterotypes, clustering human fecal metagenomic samples based on their taxonomic composition, are described as, “densely populated areas in multidimensional space of community composition” (18). Three types of enterotypes are traditionally reported, which are independent of age, gender, cultural background, and geography. We found that ET1 only appears in the SAP group (22%), and ET2 was predominant in the non-SAP group (85%), with a high abundance of *Prevotella* (**Figures S4C, D** **)**. The important genera in different enterotypes are shown in **Figure S4E**. Moreover, we found that the relative abundances of *Enterococcus*, *Parabacteroides*, *Lachnospira*, *UCG_004*, and *Clostridium_innocuum_group* were higher in the patients with ICH after developing pneumonia than those before developing pneumonia **(** **Figure S4F, G**), which indicated that *Enterococcus* and *Parabacteroides* could be sensitive biomarkers in the prediction of which patients with ICH develop stroke-associated pneumonia.

### Altered cytokine responses in ICH patients with alterations in taxonomic compositions of the gut microbiota

**Figure 5:**
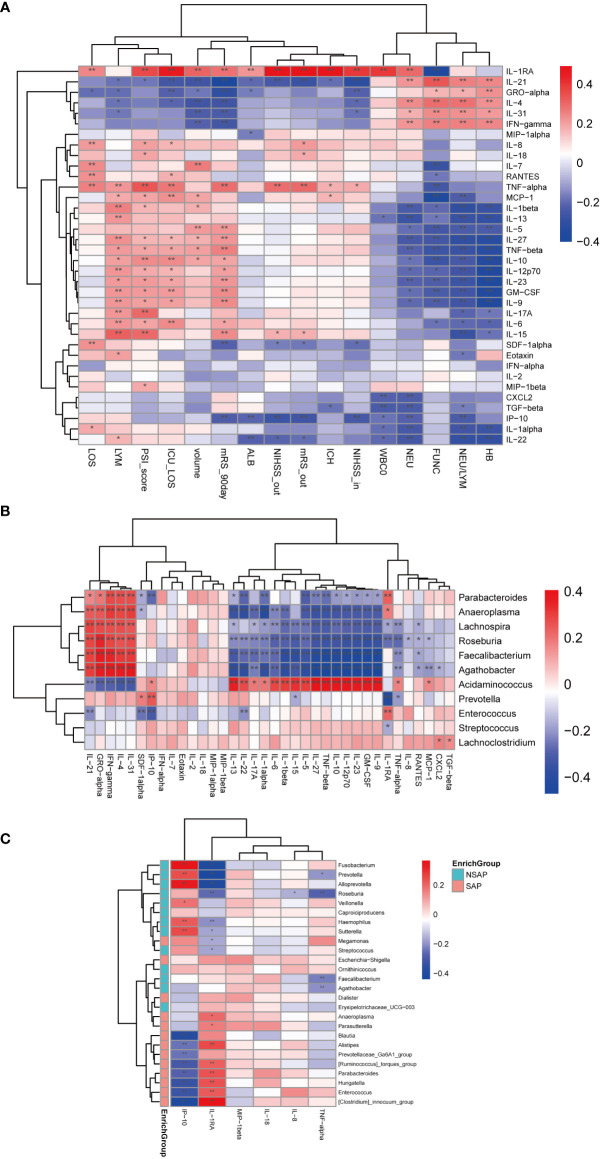
Correlations among abundances of significant fecal microbiota, clinical indexes, and serum levels of cytokines. **(A)** Heatmap of Spearman’s rank correlation coefficient among cytokines and specific clinical indexes. **(B)** Heatmap of Spearman’s rank correlation coefficient among cytokines and significant genera between ICH and control groups. **(C)** Heatmap of Spearman’s rank correlation coefficient among significant cytokines and significant genera between the SAP and non-SAP groups. *: P < 0.05. **: P < 0.01.

Gut microbial dysbiosis is associated with abnormal immune responses and is often accompanied by abnormal production of inflammatory cytokines (19). Thus, we investigated the dynamic changes of a broad spectrum of cytokines in the process of ICH and assessed the relationship between cytokine responses and clinical characteristics. Levels of different serum cytokines in the process of ICH were shown in **Figure S5**. There were 12 cytokines changed significantly at the four different time points (Kruskal-Wallis Test, Eotaxin: P = 0.036; GM-CSF: P = 0.006; IL-8: P = 0.027; IL-9: P = 0.011; IL-10: P = 0.030; IL-12p70: P = 0.014; IL-15: P = 0.006; IL-23: P = 0.015; IL-1RA: P = 0.003; IP-10: P < 0.0001; RANTES: P = 0.028; and TNF-α: P = 0.012). Among these cytokines, we found that levels of GM-CSF, IL-12p70, IL-15, IL-1RA, IL-9, IL-23, and TNF-α were gradually increased with the prolonging of time, and levels of these cytokines were positively associated with the 90-day unfavorable outcomes (**Figure 5A**). Moreover, levels of IL-10 were gradually decreased with the prolonging of time despite a slight increase at the phase T2. Levels of IP-10 decreased sharply from phase T1 to T2 and increased from phase T2 to T4. Decreased IL-10 levels and increased IP-10 levels were negatively associated with the 90-day unfavorable outcomes (**Figure 5A**). Further, IL-1RA levels were also positively related with the severity of ICH (admission and discharge NIHSS (r = 0.401 and r = 0.482, P < 0.001), PSI score (r = 0.360, P < 0.001), ICH score (r = 0.476, P < 0.001), hematoma volume(r = 0.309, P < 0.001), length of hospital stay (r = 0.244, P = 0.003) and length of ICU stay (r = 0.459, P < 0.001), white blood cell and neutrophil counts (r = 0.395 and r = 0.303, P <0.001), and poor functional outcomes of ICH (discharge and 90-day mRS scores(r = 0.492 and r = 0.285, P < 0.001)) (**Figure 5A**). These findings suggested that IL-1RA may be a strong cytokine to predict the severity and poor functional outcomes of ICH. Next, we investigated whether specific cytokine responses correlated with the relative abundance of important genera. As **Figure 5B** shows, *Enterococcus* was positively related to IL-1RA (r = 0.229, P = 0.003) and negatively related to IP-10 as well as SDF-1α (r = -0.315, P <0.001 and r = -0.253, P = 0.001). Conversely, *Prevotella* was negatively corrected with IL-1RA (r = -0.427, P < 0.001) and positively related to IP-10 and SDF-1α (r = 0.248, P = 0.001 and r = 0.182, P = 0.019). Interestingly, *Parabacteroides*, *Anaeroplasma*, *Lachnospira*, *Roseburia*, *Faecalibacterium*, and *Agathobacter* were negatively associated with proinflammatory cytokines such as IL-1α, IL-β, IL-6, TNF-β, and others, which indicated that ICH onset was accompanied by a great alteration in the intestinal microbiota and immune responses.

To further examine the cytokine responses in SAP subjects, we found that there were six cytokines statistically significant between the SAP group and non-SAP group (Wilcoxon rank-sum test, SAP *vs* non-SAP, mean [SEM], IP-10:19.9 ± 3.8 *vs*. 22.54 ± 3.181, P = 0.006; IL-8: 20.53 ± 3.7 *vs*. 13.09 ± 4.675, P = 0.014; IL-1RA: 1087 ± 91.37 *vs*. 376.3 ± 74.39, P < 0.0001; TNF-α: 3.261 ± 0.316 *vs*. 2.785 ± 0.393, P = 0.045; MIP-1β: 111.1 ± 10.15 *vs*. 70.34 ± 7.494, P = 0.013; IL-18: 10.67 ± 0.947 *vs*. 8.098 ± 1.141, P = 0.031) (**Figure S6**). Among these cytokines, IP-10 was negatively related to *Enterococcus* and Parabacteroides (r = -0.324 and r = -0.274, P < 0.001), and positively correlated with *Prevotella* (r = 0.242, P = 0.002). IL-RA, on the contrary, was positively correlated with *Enterococcus* and Parabacteroides (r = 0.219, P = 0.005 and r = 0.231, P = 0.003), and negatively related with *Prevotella* and *Roseburia* (r = -0.440 and r = -0.259, P < 0.001) (**Figure 5C**).

### *Enterococci* are promising factors in the prognosis of cerebral hemorrhage

**Figure 6:**
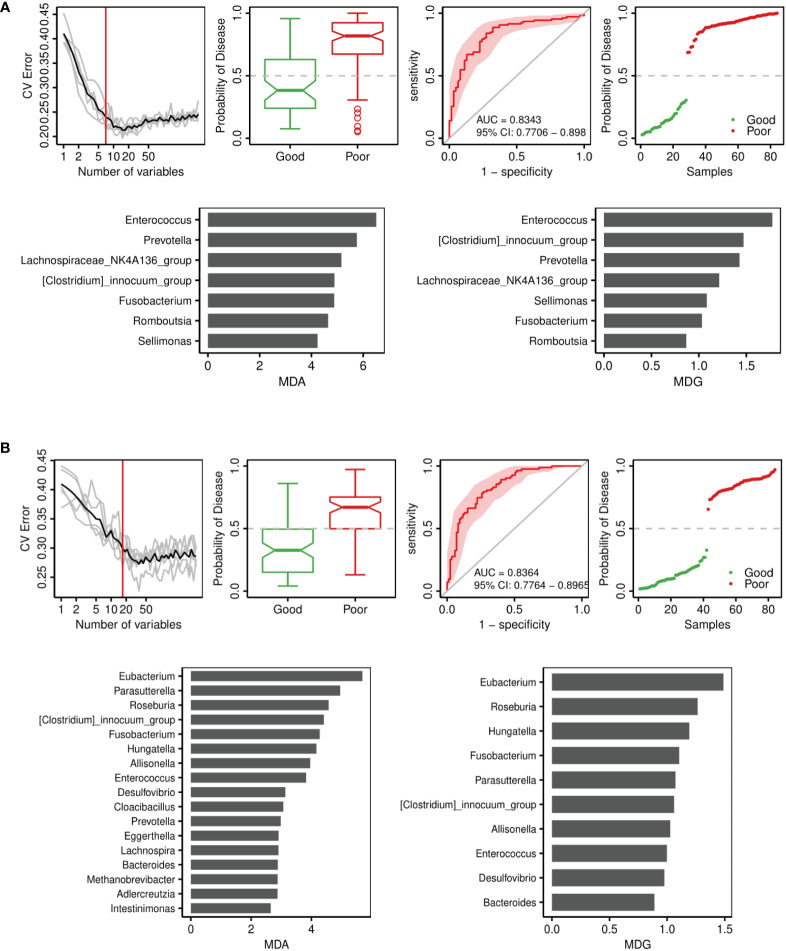
Disease classification based on gut microbiota signature. Receiver operating characteristic curve (ROC) analysis of the sensitivity and specificity of the differentially abundant genera as prognosis factors for ICH. Variable importance in random forests considering the mean decrease in accuracy (MDA) (left) or mean decrease in Gini index (MDG) (right). **(A)** Model of 14-day outcome (discharge NIHSS) of ICH. **(B)** Model of 90-day outcome (90-day mRS) of ICH.

To determine signature bacteria that could discriminate the good or poor functional outcomes of short-term and long-term prognosis, we incorporated robust statistical analysis and applied 5-fold cross-validation together with random forest to create classification models with consideration of the lowest error rate and standard deviation. The random forest model was used to select important genera. As **Figure 6A** shows, the combination of *Enterococcus*, *Prevotella*, *Lachnospiraceae_NK4A136_group*, *[Clostridium]_innocuum_group*, *Fusobacterium*, *Romboutsia*, and *Sellimonas* could distinguish the good or poor outcomes of short-term prognosis (discharge NIHSS score decrease > 40% for good outcome), with an AUC of 0.8343 (95% CI = 0.7706–0.898). Among these genera, *Enterococcus* was the major genus in the signature biomarkers’ random seed, which indicated that *Enterococcus* was likely to predict the short-term outcome of ICH. Moreover, 17 genera including *Eubacterium*, *Roseburia*, *Fusobacterium*, *Enterococcus*, and *Prevotella* consisted of the random forest of long-term outcomes of ICH (90-day mRS ≤ 2 for good outcome), with an AUC of 0.8364 (95% CI = 0.7764–0.8956) (**Figure 6B**).

## Discussion

Previous studies have shown that commensal microbiota played a critical role in degenerative and autoimmune diseases of the central nervous system (20, 21). Stroke itself markedly affects the composition of intestinal microbiota and these changes, in turn, can determine stroke outcome (9). Few studies have been conducted to reveal the characteristics of the intestinal microbiota and peripheral immunity associated with ICH *in vivo*. Here, we reported that ICH induced gut microbiota dysbiosis, which was similar to previous studies on other acute CNS injuries (9, 11, 22). Additionally, we described the cytokine response after ICH and its relationship to the intestinal microbiota.

Our study revealed the richness and diversity of fecal microbiota were altered in patients with ICH, in contrast to those in the HC group and CHD group. The microbiota structures were different between the ICH and control groups. These observations were consistent with a previous study that suggested the gut microbiota could be altered in ICH (22). In terms of the composition of the gut microbiota, we determined certain specific changes in the composition of the bacterial genera in patients with ICH relative to controls by applying the LEfSe algorithm. We observed a significant increase in putative pathobionts in the ICH group. *Enterococcus*, a genus of *Firmicutes* phylum, are considered commensal organisms of the human gastrointestinal tract. However, they can also be pathogenic, usually causing urinary tract infection, bacteremia, endocarditis, burn and surgical wound infections, neonatal sepsis, abdomen and biliary tract infections, and root canal failure (23–25). In our study, *Enterococci* levels were higher in the ICH samples, and this genus has been associated with producing bacteriocins, which are linked to mobile elements (24). Additionally, *Enterococci* are an important clinical cause of bloodstream infection. The incidences of *E. faecalis* and *E. faecium* bloodstream infections were 4.5 and 1.6 per 100000, respectively, in a population-based study (26). The researchers showed that *E. faecium* infections were associated with gastrointestinal illness and affected patients who were invalid and hospitalized, leading to a high mortality rate. Other studies showed that *Parabacteroides* was also abundant in patients with hypertension (27) and large artery atherosclerotic stroke or transient ischemic attack (28). Similarly, the less studied *Acidaminococcus* was enriched in hypertension subjects in other cohorts as well (29–31). A higher abundance of *Lachnoclostridium* could lower circulating levels of acetate, resulting in increased visceral fat negatively impacting obesity and type 2 diabetes (32). *Lachnoclostridium* has been found to produce trimethylamine (32). Trimethylamine N-oxide (TMAO) promotes atherosclerosis and is linked to platelet hyperreactivity and inflammation, which in turn participates the development of stroke and its secondary consequences (33). *Streptococcus* was found to cause neurological damage by producing neurotoxins such as streptomycin, streptodornase, and streptokinase (34). The abnormal increase of these putative pathobionts could produce endotoxins and neurotoxins and were associated with high-risk factors of ICH, which may have contributed to the development of ICH pathogenesis (34). The three main depleted genera in the ICH group, *Prevotella*, *Faecalibacterium*, and *Roseburia*, are major commensal or beneficial microbes. *Prevotella* are linked to a plant-rich diet composed of carbohydrates and fiber; although, in the gut, they have been linked to inflammatory conditions (35, 36). One study found that subjects with a high *Prevotella* abundance lost more weight when eating ad libitum whole-grain diets, suggesting *Prevotella* may control body weight (37).*Faecalibacterium* and *Roseburia* have been widely considered critical butyrate acid-producing beneficial bacteria (38, 39), whose populations were depleted in many diseases (29, 40, 41). Among these genera, our study also showed that the *Enterococcus* and *Parabaceroides* populations increased in the ICH group, and had a robust correlation with the severity of disease, inflammatory conditions, and poor outcomes. However, some beneficial microbes, such as *Prevotella* and *Roseburia*, correlated inversely with the above-mentioned factors.

Recently, studies on SAP have increased significantly. SAP is the major complication of ICH and has high mortality and morbidity (42). However, there have been few advancements in the prevention and treatment of SAP (43). Increasing evidence has shown that gut microbiota played an essential role in post-stroke infection (44, 45). Therefore, in this study, we explored the microbiota community of SAP after ICH. We found that more patients with moderate to severe ICH were admitted to the intensive care units of our clinical centers because of high SAP rates. We then found that there were structural differences in the gut microbial communities between the SAP and non-SAP groups. Consistent with the altered gut microbiota in the ICH patients, *Enterococcus* enrichment and *Prevotella* depletion were also found in the SAP patients, and *Enterococcus* was positively associated with the severity of ICH and SAP and poor outcomes of ICH, whereas we found that *Prevotella* was inversely associated. This suggested that *Enterococcus* enrichment and *Prevotella* depletion not only promoted the progression of ICH but also increased the occurrence of SAP. Similar to previous studies, *Prevotella* was associated with a reduced risk of hospital-acquired pneumonia in adult intensive care unit patients (46) and was reduced in the oropharynx of adults and children with asthma or chronic obstructive pulmonary disease (47). *Enterococcus* was similarly abundant in SAP following acute ischemic stroke (48) and acquired immune deficiency syndrome (49), which showed that *Enterococcus* could be related to stroke-induced immunodepression, a leading mechanism of SAP (50–53). Furthermore, enterotypes could be the potential predictors of SAP, as showed that ET1 was the best indicator in the SAP group driven by *Enterococcus*, and ET2 was dominant in the non-SAP group with a high abundance of *Prevotella*. However, our cohort was composed of a small population from the Southern and Northeast regions of China.

In terms of the potential microbial functions, our study showed overgrowth of facultatively anaerobic and mobile element-containing bacteria and the decrease of anaerobic bacteria in patients with ICH, which indicated that the alteration to the gut microbiota may be involved in the development of brain injury. Interestingly, the *Enterococcus* (facultatively anaerobic) increase and the *Prevotella* (anaerobic) depletion were in agreement with our findings. Further, we found that metabolic pathways of peptidoglycan biosynthesis and hexitol fermentation to lactate, formate, ethanol, and acetate had a positive association with Enterococcus in the ICH. Peptidoglycan is an essential molecule in the cell wall of both gram-positive and gram-negative bacteria. In a previous study, intraperitoneal injection of 2E7, to neutralize circulating peptidoglycan, suppressed the development of autoimmune arthritis and experimental autoimmune encephalomyelitis in mice (54), which indicated that peptidoglycan could be related to the development of autoimmune disease (55, 56). Peptidoglycan has been found in human atherosclerotic lesions (57). Increased baseline levels of peptidoglycan recognition protein-1 (PGLYRP-1), a pro-inflammatory molecule that binds peptidoglycan, were independently associated with an increased risk of first atherosclerotic cardiovascular disease (ASCVD) in a ten-year cohort, suggesting that PGLYRP-1 may contribute to the development of ASCVD (58). Cerebral ischemia is a contributing mechanism to secondary injury after ICH. Lactate accumulation induced by ischemic damages was observed in the ICH model (59, 60). Lactic acid has been shown to exacerbate ischemic brain injury by activating G protein-coupled receptor 81 (GPR81) and inhibition of GPR81 attenuated the ischemic injury (61). Additionally, early elevated cerebral lactate levels in extracellular fluid were associated with the occurrence of pneumonia in patients with aneurysmal subarachnoid hemorrhage, which may result from systemic hypoxemia or lactatemia with a damaged blood-brain barrier (62). Further, lactate accumulation in the colon could alter gut microbiota composition (63) and modulate immune responses (64). In our study, *Lactobacillales* (data not shown), *Enterococcus*, and *Streptococcus* (lactic acid bacteria) were significantly enriched in the ICH group compared to the HC group. These results suggested that microbiota-derived lactate may participate the secondary injury after ICH and increase the occurrence of SAP.

A growing body of evidence has suggested that intestinal microbes modulated the induction, training, and function of immune system responses, with gut microbiota dysbiosis related to several autoimmune and immune-mediated inflammatory diseases (65–67). Therefore, we investigated the dynamic changes of a broad spectrum of cytokines following ICH and evaluated the relationship between inflammatory cytokine response and long-term outcomes of ICH and signature microbiota. In our study, we found that Eotaxin, GM-CSF, IL-8, IL-9, IL-10, IL-12p70, IL-15, IL-23, IL-1RA, IP-10, RANTES, and TNF-α were changed significantly in the progression of ICH. levels of GM-CSF, IL-12p70, IL-15, IL-1RA, IL-9, IL-23, and TNF-α were increased and levels of IL-10 decreased gradually, which positively correlated with 90-day poor outcomes. Many studies revealed that GM-CSF promoted leptomeningeal collateral growth, decreased the infarct size, and improved long-term functional outcomes in the experimental stroke (68, 69). GM-CSF was more than a growth factor and researchers showed that GM-CSF also promoted neuroinflammation by increasing LPS-induced production of proinflammatory mediators (70). In line with previous study, astrocyte-derived IL-15 significantly increased in the ICH patients and experimental ICH and aggravated brain injury following ICH through the proinflammatory response amplification of microglia in the setting of ICH (71). Similarly, astrocytic IL-15 also exacerbates brain damage after ischemic stroke by enhancing cell-mediated immune responses (72). Researchers found that IL-23 signaling could promote Th2 polarization and enhance Th2 expression in allergic inflammation (73). Expression of IL-23 and IL-17 increased in sequence following ICH and IL-23/IL-17 axis promoted secondary brain injury in ICH model mice (74). However, IL-17 levels did not increase in the acute phase in our study, which indicates that IL-23 may influence ICH in a Th17-independent manner. Consistent with previous research, higher IL-1RA, erythrocyte sedimentation rate, and CRP were correlated with dependent stroke outcome (mRS >3) in acute ischemic stroke (75). Moreover, IL-5, IL-6, IL-9, and IL-27 were also positively correlated with long-term functional outcomes. Increased serum levels of IL-6 and IL-10 were detected in intraparenchymal hemorrhage (76), and higher admission IL-6 levels were associated with unfavorable 90-day functional outcomes and hematoma and perihematomal edema volumes (77). Additionally, IL-6 and IL-10 levels were higher in hemorrhage stroke patients with 1-month unfavorable outcomes (78). Hematoma expansion is a major cause of morbidity and mortality after ICH, and inflammation may be associated with its pathogenesis. Higher plasma IL-10 levels were related to the hematoma expansion in spontaneous ICH and worse 30-day outcomes (79). However, a study on IL-10-/- mice showed that the presence of IL-10 was protective against the development of ICH (80). Although IL-10 is regarded as an anti-inflammatory cytokine to prevent inflammatory and autoimmune pathologies by limiting the immune response to pathogens (81), it also exhibits proinflammatory activities. A study showed that IL-10 treatment stimulated lipopolysaccharide (LPS)-induced release of IFN-γ and enhanced activation of CTL and NK cells after LPS injection, though IL-10 treatment upon LPS-induced IFN-γ release could not be reproduced in whole blood *in vitro* (82). IL-5, IL-9, IL-10, IL-23, and IL-27 are also related to the Th2 response (T-cell response associated with allergies, progressive systemic sclerosis, and autoimmune disorders) (83). Researchers observed that IL-27 was upregulated centrally and peripherally after ICH, and IL-27 treatment improved ICH outcomes by reducing edema and increasing iron and hematoma clearance (84). However, higher IL-27 levels were correlated with poor 90-day outcomes in our results. The findings above suggested that high-dose anti-inflammatory therapy in patients with inflammatory disorders could be associated with undesired proinflammatory effects *in vivo*.

In contrast, IL-21, CXCL1, IL-4, IL-31, IFN-γ, SDF-1α, and IP-10 were negatively associated with 90-day unfavorable outcomes. Recently, IL-4/STAT6 signaling accelerated microglia-and macrophage-mediated hematoma clearance and improved neurofunctional recovery following ICH in blood and collagenase injection models (85). Additionally, in a study about the relationship between ex vivo cytokine synthesis and 3-month outcomes after ischemic stroke, decreased release of IP-10, TNFα, IL-1β, and IL-12; increased release of IL-10 and IL-8; and higher plasma IL-6 levels were associated with poor outcomes (86). Additionally, decreased release of IP-10 and TNF-α after ex vivo blood stimulation with endotoxin was associated with poor outcomes after stroke, suggesting that the inhibition of both the MyD88-dependent and MyD88-independent pathways of toll-like receptors (TLR)4 signaling in blood cells was associated with poor prognosis in stroke patients (87). Reduced IFN-γ production caused by impaired NK and T cell response was the crucial stroke-induced defect in the antibacterial defense. IFN-γ supplementation effectively inhibited bacterial infections after stroke (50). Neovascularization after ICH is an important compensatory response that mediates brain repair and improves the clinical outcome. The Tp53 Arg72Pro single-nucleotide polymorphism increased endothelial cell survival and triggered efficient endothelial progenitor cell mobilization *via* vascular endothelial growth factor and SDF-1α, resulting in neovascularization after experimental ICH (88). In conclusion, the onset of ICH induced massive, rapid activation of the peripheral immune system and Th2 responses were correlated with worse 90-day outcomes.

Furthermore, *Enterococcus* was positively associated with IL-RA and negatively associated with IP-10 and SDF-1α, while *Prevotella* showed an inverse association. Peptidoglycan is detected by multiple pattern-recognition receptors and triggers inflammatory responses in immune and nonimmune cells (89). TLR2s are known to be the signaling receptors for peptidoglycan, which induced IL-1RA gene expression by activating the p38 stress-activated protein kinase (90). IP-10 has been shown to have direct antibacterial activity similar to α-defensins, like against *Escherichia coli* and *Listeria* monocytogenes (91). In addition, IFN-γ signaling in enteric glia cells (EGCs) maintains intestinal homeostasis and immunity and improves tissue repair after intestinal damage caused by pathogen infection. Researchers have identified IP-10 as the critical response cytokine in IFN-γ signaling, thus the IFNγ–EGC–IP-10 axis is essential to the immune response and tissue repair after infectious challenge (92). Collectively, this evidence showed that *Enterococcus* interacted with cytokines such as IL-1RA, IP-10, and SDF-1; promoted the TLR-2 pathway; inhibited the TLR-4 pathway (87); induced neovascularization; and disturbed the homeostasis of the intestinal microbiota to aggravate the inflammatory response and worsen ICH outcomes.

Stroke-induced immunosuppression (SIIS) was characterized by decreased lymphocyte counts in the spleen, blood, and thymus; impaired early NK and T cell responses, and a shift from Th1 to Th2 (50). This syndrome increased the susceptibility to stroke-associated infections. Among these infections, SAP was the major acute type of ICH and can worsen ICH functional outcomes (93). To elucidate the molecular mechanisms of SAP, the peripheral suppression of the immune system after the occurrence of ICH must be considered. In this study, we found that there were six cytokines that were significantly correlated with SAP, including IP-10, IL-1RA, TNF-α, MIP-1β, IL-18, and IL-8. IP-10 was the only cytokine that was decreased in the SAP group. IFN-γ plays a pivotal role in preventing bacterial infections after stroke. Studies have revealed that supplementing with IFN-γ by adoptive transfer of IFN-γ–producing lymphocytes or recombinant IFN-γ treatment inhibited bacteremia and pneumonia (50). However, this did not prove whether the downstream effector of IFN-γ was associated with stroke-associated infections or not. IP-10, also called IFN-γ-inducible protein 10, is a chemokine secreted from cells stimulated with type I and II IFNs and LPS (94). It is vital in controlling pneumonia by enhancing IFN-γ production and reinforcing leukocyte antibacterial responses (95). In a previous study, at the early stage of Klebsiella administration, anti-IP-10 antibody treatment led to 10- to 100-fold increases in the number of Klebsiella pneumoniae CFU isolated from lung homogenates compared to IgG administration. Additionally, adenovirus-mediated expression of IP-10 led to 30- to 100-fold reductions in lung and blood CFU in Klebsiella-infected mice in the early stage (95). Therefore, the IFN-γ-IP-10 axis may be a candidate pathway for immunotherapy of SAP or severe respiratory tract infection. Decreased secretion of TNF-α and IFN-γ has contributed to spontaneous bacterial infections. However, a reduction of endotoxin-induced TNF-α was observed 12 h and 2 d after middle cerebral artery occlusion and returned to control levels on day 5 (50). Our study showed that TNF-α levels were increased in the SAP group, which suggested that the increase of TNF-α present during the late stage of stroke could also be linked to SAP, as the duration of SIIS still remained unknown. Stroke severity was the most important predictor of infection risk, and increased plasma IL-1RA levels were independently associated with infection risk after adjusting for stroke severity. This suggested that IL-1RA was a strong predictor of post-stroke infection (96). Moreover, in a previous study, the A2A2 genotype of the IL-1RA gene was associated with the risk of adverse outcomes of severe community-acquired pneumonia in Indian children (97), though there were some different findings in other studies (98, 99). MIP-1β, an inflammatory chemokine, has an impact on vasculopathy. Researchers have found that MIP-1β inhibition improved endothelial progenitor cell (EPC) function and enhanced EPC homing and ischemia-induced neovasculogenesis (100). Increased IL-18 and IL-8 expressions contributed to the development and severity of stroke (101–103) and IL-18 also participated in hypoxic-ischemic brain injury (104). Interestingly, a novel innate immunity pathway consisting of lipoteichoid acid, produced by gram-negative bacteria, was sensed by the NLRP6 inflammasome and exacerbated a systemic gram-positive pathogen infection *via* the production of IL-18 (105). Unexpectedly, IL-18 was not associated with the genus *Enterococcus* in our study. Further analysis showed an association between the cytokines and genera and revealed that increased bacteria in the SAP group, especially *Enterococcus*, enhanced the expression of IL-1RA and decreased IP-10 levels to promote SAP. Increased bacteria in the non-SAP group, particularly *Prevotella*, were inversely related to SAP.

Random forest analysis showed that *Enterococci* were the critical biomarkers in determining either the good or poor functional outcomes of short-term and long-term prognoses. Notably, *Enterococci* were the most important biomarkers in predicting short-term functional outcomes, which was due to its levels gradually increasing throughout the ICH process and peaking at phase T4.

The major findings of our study were that the gut microbiota changed dynamically throughout the duration of ICH, and gut dysbiosis with *Enterococcus* enrichment and *Prevotella* depletion not only promoted ICH but also SAP. Moreover, we investigated the dynamic changes of a broad spectrum of cytokines in the process of ICH and confirmed the roles of these cytokines in patients with ICH, and examined the relationship between genera and cytokine responses. Our study does have some limitations. First, the age, gender, and comorbidities of the ICH group and the control groups were not identical, although a generalized linear model was applied to control the possible confounding factors. Additionally, the stool samples in different phases of the ICH process were varied, as ICH could reduce gastrointestinal motility (22). Second, short-chain fatty acid (SCFA) levels, which potentially mediate gut-brain communication, were not tested (106), although a number of studies have revealed that SCFAs played a beneficial and anti-inflammatory role in stroke (107, 108). Third, antibiotics are unavoidable, important factors for ICH patients and gut microbiota. ICH patients in this study were recruited from intensive care units and 73.4% of them were diagnosed with pneumonia within 7 days of ICH. Thus, we should consider the effect of antibiotics on intestinal microbiota. However, the impact of different types of antibiotics and their application times on intestinal flora *in vivo* remains unknown, and it is difficult to control the antibiotics used in unpredictable medical conditions. Finally, for the study scale, we did not analyze the correlations between every taxon and every cytokine tested in this study under the consideration of the limited statistical power of multiple comparisons. Consequently, the related changes in the microbiota and serum cytokines were analyzed under the assumption that the altered microbiota may trigger peripheral inflammatory responses that contributed to ICH or SAP. Additionally, the specific mechanisms underlying the microbiota and ICH process were not explored in this study. Therefore, in a future study, we plan to simulate the intestinal alteration by enriching *Enterococcus* or depleting *Prevotella* in experimental ICH to verify the potential targets and elucidate their causal relationship in the gut-brain axis. Moreover, specific immune responses stimulated by a particular species, or a group of gut microorganisms, need to be investigated. We will analyze the dynamic changes in cytokine responses in patients with SAP in our subsequent studies to identify the changes in inflammatory responses. More study patients will be enrolled in the future to support our findings. As discussed, SIIS was a key mechanism of ICH and SAP. More information about SIIS including the duration, the cytokine storm, and its activation is needed.

## Conclusion

In summary, to the best of our knowledge, this is the first study to show that patients with *Enterococcus* enrichment and *Prevotella* depletion in the gut microbiota had increased risk of ICH and SAP *in vivo*. Changes in a broad spectrum of cytokines associated with the signature microbiota proved that microbiota alterations with aberrant host immune responses were related to ICH pathogenesis. Elucidation of the interaction between intestinal microbiota and the peripheral immune response would help to understand ICH pathogenesis. The altered gut microbiota composition and serum cytokine profiles are potential biomarkers that reflect the inciting physiologic insult/stress involved with ICH. Gut microbiota modulation may help to the development of intervention strategies targeting microbiota dysbiosis for ICH.

## Data availability statement

The original contributions presented in the study are publicly available. This data can be found here: https://www.ncbi.nlm.nih.gov/bioproject/PRJNA805052.

## Ethics statement

The studies involving human participants were reviewed and approved by all participating centers. The patients or volunteers provided their written informed consent to participate in this study.

## Author contributions

JG, LZ, JZ, JL and YC designed the study and provided guidance on the data analysis and interpretation/presentation of the data. JG is the subject primarily responsible for the clinical trial and provided a critical review of the manuscript. JL, YC, and XS performed and analyzed all the data. JL drafted most sections of the manuscript. GT, JZ, ZL, XY, TH, GH, LW and YH conceived the study, supervised the participants, and revised the manuscript. CW and BW participated in specimen processing and quality control. YZ, QG, BG, ML, RW, JY and WC organized and managed the study including samples and data collection, and quality assurance. All authors contributed to the article and approved the submitted version. JL and YC have an equal contribution to this manuscript.

## Funding

The present study was funded by the National Natural Science Foundation of China (grant number:81974559), Key Research and Development Project of Guangdong Province (grant number: 2020B1111100009), Natural Science Foundation of Guangdong Province, China (grant number: 2020A1515010992), the Special Research Project of Guangdong Provincial Hospital of Chinese Medicine (grant number: YN2018ML06), State Key Laboratory of Dampness Syndrome of Chinese Medicine (grant number: SZ2021ZZ06), Key-Area Research and Development Program of Guangdong Province (grant number: 2020B1111100010), and Double First-Class” and High-level University Discipline Collaborative Innovation Team Project of Guangzhou University of Chinese Medicine (2021xk48). Guangdong Provincial Key Laboratory of Research on Emergency in TCM (grant number: 2017B030314176).

## Acknowledgments

We thank the patients or participants who volunteered for this study. We thank Xiaodong Fang and Zhang Wang for technical and logistical assistance, and Yu Yan, Zhixiang Yan, and Shuang Zhao for discussions. Further, we also would like to give thanks for the support of the Biological Resource Center of The Second Affiliated Hospital of Guangzhou University of Chinese Medicine and Guangdong Provincial Key Laboratory of Clinical Research on Traditional Chinese Medicine Syndrome for specimen processing and quality control.

## Conflict of interest

The authors declare that the research was conducted in the absence of any commercial or financial relationships that could be construed as a potential conflict of interest.

## Publisher’s note

All claims expressed in this article are solely those of the authors and do not necessarily represent those of their affiliated organizations, or those of the publisher, the editors and the reviewers. Any product that may be evaluated in this article, or claim that may be made by its manufacturer, is not guaranteed or endorsed by the publisher.

## Abbreviations

16S rRNA, 16S ribosomal RNA; ICH, intracerebral hemorrhage; HC, healthy control; CHD, coronary heart disease; SAP, stroke-associated pneumonia; CDC, Centers for Disease Control and Prevention; SD, standard deviation; IQR, interquartile range; ANOVA, analysis of variance; ROC, receiver operator characteristic; PCoA, principal coordinate analysis; PERMANOVA, permutational multivariate analysis of variance; LDA, linear discriminant analysis; LEfSe, linear discriminant analysis effect size; GLM, generalized linear model; NAD, nicotinamide adenine dinucleotide; MDA, mean decrease in accuracy; MDG, mean decrease in Gini index; PGLYRP-1, peptidoglycan recognition protein-1; LPS, lipopolysaccharide; TLR, toll-like receptors; SIIS, Stroke-induced immunosuppression; EGCs, enteric glia cells; EPC, endothelial progenitor cell; ASCVD, atherosclerotic cardiovascular disease; GPR81, G protein-coupled receptor 81.
