# Supplementary material for: Gut microbiota composition reflects disease progression, severity and outcome, and dysfunctional immune responses in patients with hypertensive intracerebral hemorrhage
Source: Front Immunol. 2022 Oct 14;13:869846. doi: 10.3389/fimmu.2022.869846 (PMC9699794; doi:10.3389/fimmu.2022.869846)
Supplement: Supplementary file 1 [file DataSheet_1.docx]

**Supplementary**

**Table S1.** **Clinical characteristics of the study participants.**

| Clinical characteristics | ICH group (T1) (n=64) | HC group (n=23) | CHD group (n=46) | P-value  (ICH-HC) | P-value  (ICH-CHD) |
| --- | --- | --- | --- | --- | --- |
| Male, n (%) | 38 (59.375) | 6 (26.087) | 31 (67.391) | .006** | .391 |
| Age, yr, mean (SD)/median (IQR)# | 59.880 (10.657) | 25 (6) # | 62.390 (7.597) | < 0.001*** | .151 |
| **History** |  |  |  |  |  |
| Previous strokes, n (%) | 21 (32.813) | N/A | N/A | N/A | N/A |
| Hypertension, n (%) | 49 (76.563) | 0 | 12 (26.087) | < 0.001*** | < 0.001*** |
| Current smoker, n (%) | 10 (15.625) | 0 | 9 (19.565) | .039* | .321 |
| Alcoholism, n (%) | 5 (7.813) | 0 | 11 (23.913) | .255 | .046* |
| Diabetes mellitus, n (%) | 9 (14.063) | N/A | N/A | N/A | N/A |
| CHD, n (%) | 6 (9.375) | 0 | 46 (100) | .128 | N/A |
| Obstructive pulmonary disease, n (%) | 3 (4.688) | N/A | N/A | N/A | N/A |
| **Radiology** |  |  |  |  |  |
| Hematoma location, n (%) |  |  |  |  |  |
| supratentorial | 58 (90.625) | N/A | N/A | N/A | N/A |
| infratentorial | 6 (9.375) | N/A | N/A | N/A | N/A |
| ICH volume, mL, median (IQR) | 11.2 (13.95) | N/A | N/A | N/A | N/A |
| Midline shift (MLS), n (%) | 26 (40.625) | N/A | N/A | N/A | N/A |
| **Complication** |  |  |  |  |  |
| SAP, n (%) | 47 (73.438) | N/A | N/A | N/A | N/A |
| Venous thromboembolic  events (VTE), n (%) | 8 (12.5) | N/A | N/A | N/A | N/A |
| intraventricular extension  of hemorrhage, n (%) | 27 (42.188) | N/A | N/A | N/A | N/A |
| Haematoma expansion, n (%) | 8 (12.5) | N/A | N/A | N/A | N/A |
| Gastrointestinal bleeding, n (%) | 1 (1.563) | N/A | N/A | N/A | N/A |
| Electrolyte disturbance, n (%)  **Baseline characteristics**  inpatient(h), median (IQR)  enrollment (d), median (IQR)  antibiotic (d), median (IQR)  SBP (mmHg), mean (SD)  DBP (mmHg), mean (SD)  temperature (℃), median (IQR)  respiratory rate (pm), median (IQR)  pulse rate (bpm), mean (SD)  ICH score, median (IQR)  NIHSS on admission, median (IQR)  GCS on admission, median (IQR) | 16 (25)  6 (11)  2 (1)  1 (1)  168.89 (26.968)  99.14 (15.88)  36.5 (0.3)  19 (2)  78 (18)  1 (2)  10 (9)  14 (6) | N/A  N/A  N/A  N/A  N/A  N/A  N/A  N/A  N/A  N/A  N/A  N/A | N/A  N/A  N/A  N/A  N/A  N/A  N/A  N/A  N/A  N/A  N/A  N/A | N/A  N/A  N/A  N/A  N/A  N/A  N/A  N/A  N/A  N/A  N/A  N/A | N/A  N/A  N/A  N/A  N/A  N/A  N/A  N/A  N/A  N/A  N/A  N/A |
| **Examination** |  |  |  |  |  |
| TC, mmol/L, median (IQR) | 4.45 (1.55) | N/A | 3.915 (1.3) | N/A | .132 |
| missing data, n (%) | 7 (10.938) | N/A | 4 (8.696) | N/A | N/A |
| TG, mmol/L, median (IQR) | .995 (1.135) | N/A | 1.43 (.52) | N/A | .012* |
| missing data, n (%) | 6 (9.375) | N/A | 4 (8.511) | N/A | N/A |
| LDL, mmol/L, mean (SD) | 2.595 (.886) | N/A | 2.623 (.857) | N/A | .875 |
| missing data, n (%) | 7 (10.938) | N/A | 4 (8.696) | N/A | N/A |
| HDL, mmol/L, median (IQR) | 1.245 (0.46) | N/A | 1.19 (.41) | N/A | .849 |
| missing data, n (%) | 8 (12.5) | N/A | 4 (8.696) | N/A | N/A |
| WBC, × 10^9^/L, mean (SD)  Neutrophil, × 10^9^/L, median (IQR)  Lymphocyte, × 10^9^/L, median (IQR)  NLR, median (IQR) | 10.252 (3.398)  7.945 (5.45)  1.375 (0.693)  6.099 (6.443) | N/A  N/A  N/A  N/A | N/A  N/A  N/A  N/A | N/A  N/A  N/A  N/A | N/A  N/A  N/A  N/A |
| **Outcome**  Length of stay, median (IQR)  Length of ICU, median (IQR)  Discharge NIHSS decrease >40% at 14 days, n (%)  90-day mRS score ≤2, n (%) | 18.50 (7)  5 (11)  27 (42.20)  37 (57.81) | N/A  N/A  N/A  N/A | N/A  N/A  N/A  N/A | N/A  N/A  N/A  N/A | N/A  N/A  N/A  N/A |

SD, standard deviation; IQR, interquartile range; CHD, coronary heart disease; SAP, stroke-associated pneumonia;

inpatient, time from onset to admission; enrollment, time from onset to enrollment; antibiotic, time from onset to antibiotic use; SBP, systolic blood pressure on admission; DBP, diastolic blood pressure on admission; TC, total cholesterol; TG, triglyceride; LDL, low-density lipoprotein; HDL, high-density lipoprotein; WBC, white blood cell count; NLR, neutrophil-to-lymphocyte ratio. The ICH score includes GCS score (0 points for GCS score 13–15, 1 point for GCS score 5–12, and 2 points for GCS score 3-4), age (1 point for ≥ 80 years), infratentorial origin (1 point), ICH volume (1 point for ≥ 30 cm^3^), and intraventricular hemorrhage (1 point). NIHSS, National Institutes of Health Stroke Scale; WBC, white blood cell; GCS, Glasgow Coma Scale.

*: P < 0.05, **: P < 0.01, ***: P < 0.001.

**Table S2. General linear models for fecal genera.**

**A**

| Genus | Odds Ratio | 95 % CI | P value |
| --- | --- | --- | --- |
| *Enterococcus* | 1.5065 | 0.8127-2.7927 | 0.0249* |
| *Acidaminococcus* | 1.7538 | 0.92189-3.3362 | 0.0842 |
| *Faecalibacterium* | 0.94473 | 0.50095-1.78160 | 0.708 |
| *Lachnoclostridium* | 0.90109 | 0.47844-1.69710 | 0.9318 |
| *Parabacteroides* | 0.42896 | 0.2266-0.81203 | 0.01393 * |
| *Prevotella* | 0.40819 | 0.21347-0.78053 | 0.055719 |
| *Streptococcus* | 0.17877 | 0.091794-0.34816 | 0.00023*** |
| *Veillonella* | 0.46642 | 0.24256-0.89689 | 0.00541** |
| *Clostridium_innocuum_group* | 1.198 | 0.63534-2.2588 | 0.045587* |
| Romboutsia | 2.8667 | 1.5447-5.4257 | 0.004438** |
| *Megamonas* | 0.34059 | 0.17959-0.64593 | 0.00341** |

**B**

| Genus | Odds Ratio | 95 % CI | P value |
| --- | --- | --- | --- |
| *Enterococcus* | 2.4082 | 1.0334-5.612 | 0.76383 |
| *Parabacteroides* | 1.2277 | 0.54206-2.7808 | 0.48664 |
| *Prevotella* | 2.0389 | 0.8704-4.7762 | 0.9327 |
| *Roseburia* | 0.98304 | 0.44889-2.1528 | 0.81975 |
| *Lachnoclostridium* | 2.6373 | 1.4420-6.0784 | 0.857 |
| *Eubacterium_eligens_group* | 0.17515 | 0.0755-0.4064 | 6.28e-6*** |
| *Dorea* | 0.09977 | 0.04145-0.24013 | 8.66e-5*** |
| *Lachnospira* | 0.17851 | 0.07538-0.42273 | 0.000917*** |
| *Fusicatenibacter* | 0.20522 | 0.09142-0.46068 | 0.00128** |

**C**

| Genus | Odds Ratio | 95 % CI | P value |
| --- | --- | --- | --- |
| *Enterococcus* | 1.5881 | 0.6662-3.7857 | 0.7848 |
| *Parabacteroides* | 2.3085 | 1.0237-5.2059 | 0.31574 |
| *Blautia* | 2.1825 | 0.89403-5.3279 | 0.0316* |
| *Prevotella* | 0.35078 | 0.14844-0.82894 | 0.023068* |
| *Lachnoanaerobaculum* | 0.43657 | 0.17874-1.0663 | 0.088 |
| *Acidaminococcus* | 0.402160 | 0.17283-0.93578 | 0.7736 |
| *Ruminococcus_torques_group* | 2.3251 | 0.99549-5.4305 | 0.001399** |
| *Sutterella* | 0.29214 | 0.12803-0.666620 | 0.00613** |
| *Veillonella* | 0.51662 | 0.22214-1.2015 | 0.037089* |

Result of the general linear models (GLMs) for significant genera (sequence counts) based on the group factors and possible confounding factors. Table S2A is for the ICH (n=170) and HC (n=23) groups adjusted for age, gender, hypertension alcoholism, smoke, pneumonia, and antibiotic use. Table S2B is for the ICH (n=170) and CHD (n=46) group adjusted for age, hypertension, alcoholism, pneumonia, and antibiotic use. Table S2C is for the SAP (n=143) and non-SAP (n=41) groups adjusted for age, hematoma volume, NIHSS, and antibiotic use.

*: P < 0.05, **: P < 0.01, ***: P < 0.001.

**Table S3.** Characteristics of Patients in the SAP and Non-SAP

| Clinical characteristics | Non-SAP (T1) (n=17) | SAP (T1) (n=47) | P-value |
| --- | --- | --- | --- |
| Male, n (%) | 12 (70.588) | 26 (55.319) | .272 |
| Age, yr, median (IQR) | 56 (14) | 57 (17) | .357 |
| **History** |  |  |  |
| Previous strokes, n (%) | 5 (29.412) | 16 (34.043) | .727 |
| Hypertension, n (%) | 12 (70.588) | 37 (78.723) | .497 |
| Current smoker, n (%) | 2 (11.765) | 8 (17.021) | 1.000 |
| Alcoholism, n (%) | 0 | 5 (10.638) | .313 |
| Diabetes mellitus, n (%) | 1 (5.882) | 8 (17.021) | .424 |
| CHD, n (%) | 1 (5.882) | 5 (10.638) | 1.000 |
| Obstructive pulmonary disease, n (%) | 0 | 3 (6.382) | .559 |
| **Radiology** |  |  |  |
| Hematoma location, n (%) |  |  |  |
| supratentorial | 15 (88.235) | 43 (91.489) | .652 |
| infratentorial | 2 (11.765) | 4 (8.511) |  |
| ICH volume, mL, median (IQR) | 6.54 (12.5) | 13 (21) | .007** |
| Midline shift, n (%) | 2 (11.765) | 24 (51.064) | .008** |
| **Others**  SAP onset time (days), median (IQR) | / | 2 (2) | / |
| WBC, × 109/L, median (IQR) | 6.92 (4.1) | 10.7 (4.65) | .003** |
| Neutrophil, × 109/L, median (IQR) | 4.45 (4.88) | 8.36 (4.86) | .012* |
| Lymphocyte, × 109/L, median (IQR) | 1.44 (0.78) | 1.25 (0.83) | .189 |
| NLR, median (IQR) | 3.5 (5.406) | 6.846 (6.112) | .021* |
| ICH score, median (IQR)  FUNC score, median (IQR)  PSI, median (IQR) | 0 (1)  10 (0)  / | 1 (2)  10 (2)  82 (31) | <0.001***  <0.001***  / |
| NIHSS on admission, median (IQR) | 7 (5) | 13 (12) | <0.001*** |
| GCS on admission, median (IQR) | 15 (0) | 13 (7) | <0.001*** |
| Length of stay, median (IQR)  Length of ICU, median (IQR)  Discharge NIHSS decrease >40% at 14 days  90-day mRS score ≤2, n (%) | 18 (11)  0 (5)  13 (76.471)  12 (70.588) | 18.5 (6.586)  7.5 (12)  14 (29.787)  25 (53.191) | .001***  .322  .001***  .254 |

IQR, interquartile range; CHD, coronary heart disease; SAP, stroke-associated pneumonia; NIHSS, National Institutes of Health Stroke Scale; GCS, Glasgow Coma Scale; WBC, white blood cell count; NLR, neutrophil-to-lymphocyte ratio; The ICH score includes GCS score (0 points for GCS score 13–15, 1 point for GCS score 5–12, and 2 points for GCS score 3-4), age (1 point for ≥ 80 years), infratentorial origin (1 point). The FUNC score includes ICH volume( 4 points for < 30 cm^3^, 2 points for 30-60 cm^3^, 0 point for > 60cm^3^), age(2 points for < 70 years, 1 point for 70-79 years, 0 point for ≥ 80 years),ICH location( 2 points for lobar, 1 point for deep, 0 point for infratentorial); GCS score (2 points for ≥ 9), pre-ICH cognitive impairment (1 point for no).

*: P < 0.05, **: P < 0.01, ***: P < 0.001.


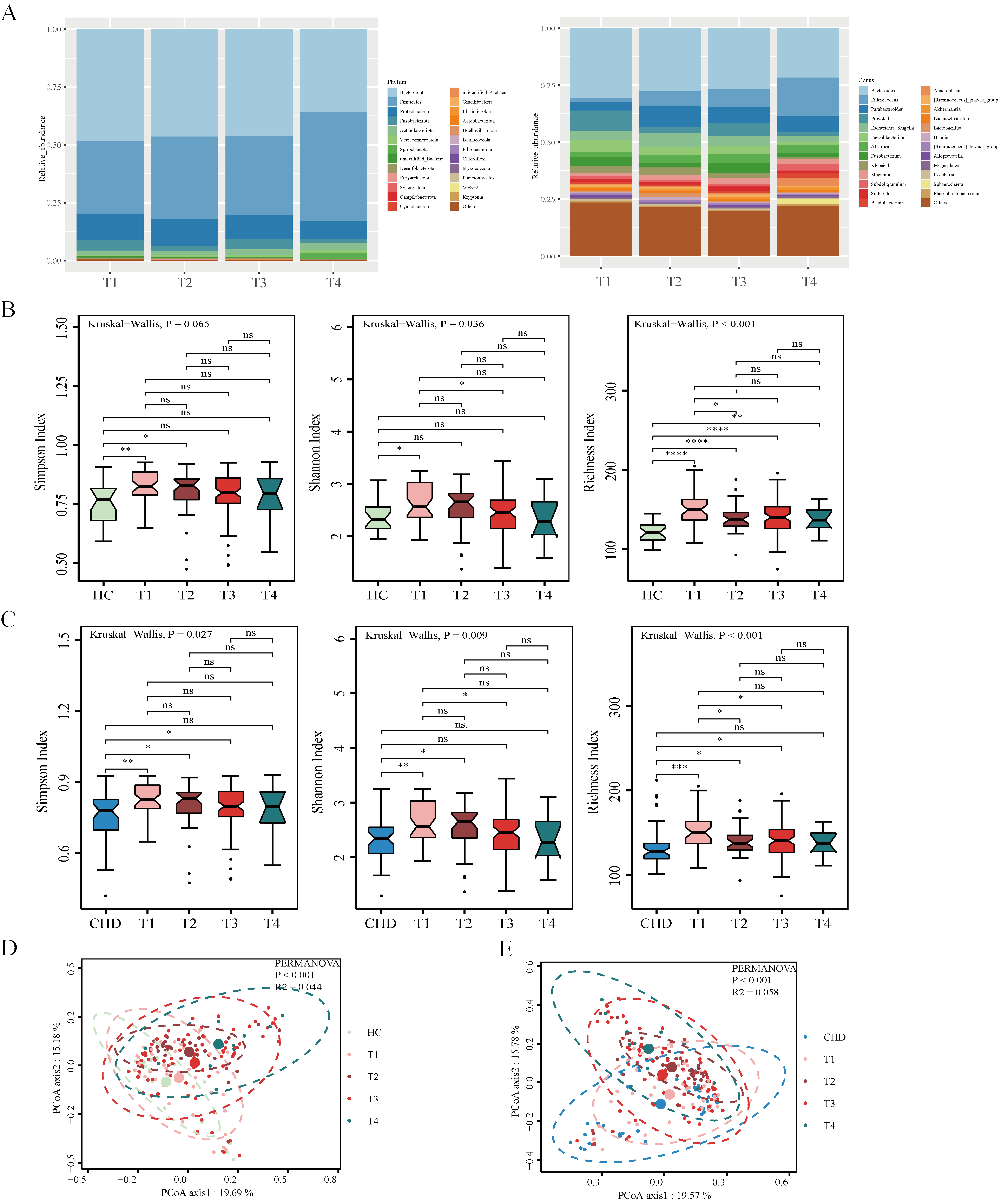


**Figure S1**. Gut microbiota changes dynamically with the prolongation of ICH. **(A)** Gut microbial composition at the phylum level and genus level. **(B-C)** Box plots depict differences in the fecal microbiota diversity indices between the ICH and control groups according to the Simpson index, Shannon index, and richness index based on OTU counts. Each box plot represents the median, interquartile range, minimum, and maximum values. OUT: operational taxonomic units. **(D-E)** PCoA with the Bray-Curtis dissimilarities showing the gut microbiota composition among healthy controls **(D)** or CHD controls **(E)** and the acute (T1–T2) and subacute (T3-T4) phases of patients with ICH. *: P < 0.05, **: P < 0.01, ***: P < 0.001, ****: P < 0.0001.


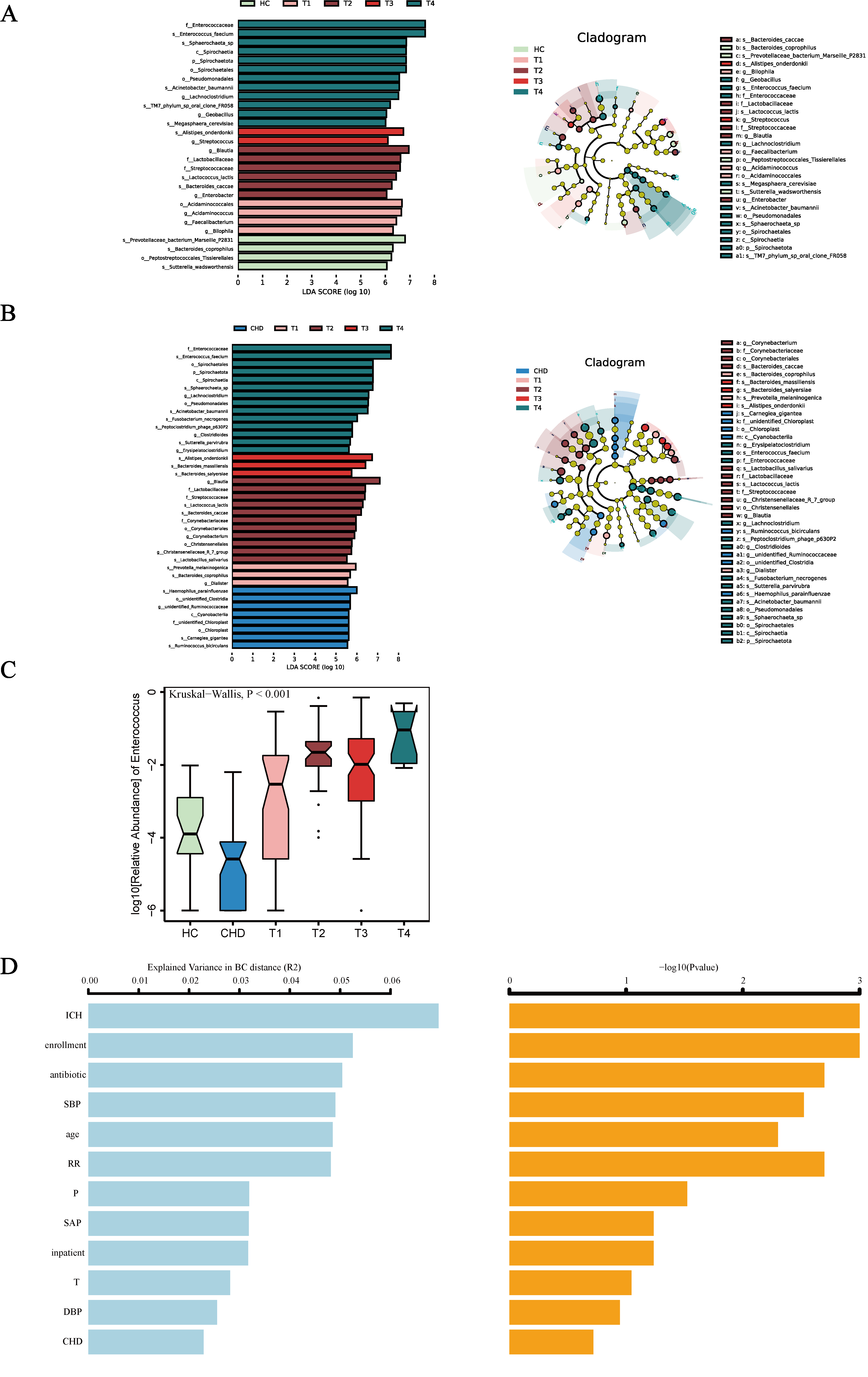


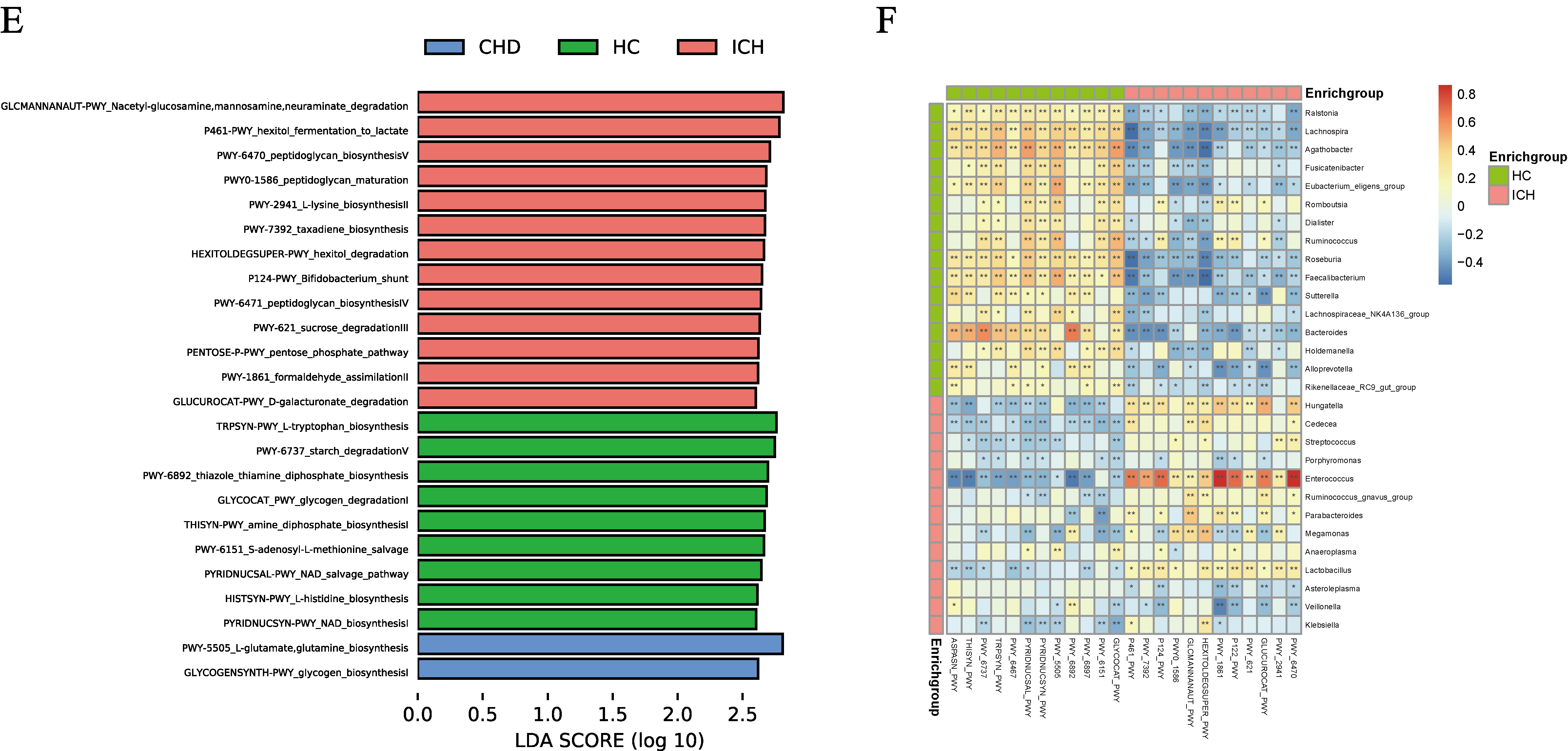


**Figure S2**. Taxonomic differences of fecal microbiota in ICH and control groups, clinical phenotypes contributing to the flora variation, and associated metabolic pathways. **(A-B)** A Linear discriminant analysis (LDA) effect size (LEfSe) analysis revealed significant bacterial differences in fecal microbiota among different phases of ICH and control groups. LDA scores (log10) > 6 **(A)**, (log10) > 5 **(B)** and P < 0.05 are shown (left panel). Cladogram using the LEfSe method indicating the phylogenetic distribution of fecal microbiota associated with ICH and control participants (right panel). **(C)** The relative abundance of *Enterococcus* was significantly higher in the patients with ICH in different phases than in control groups (P < 0.001). **(D)** The top 12 clinical phenotypes contribute to the flora variation by using permutational multivariate analysis of variance. The ICH score includes GCS score (0 points for GCS score 13–15, 1 point for GCS score 5–12, and 2 points for GCS score 3-4), age (1 point for ≥ 80 years), infratentorial origin (1 point). enrollment: time from onset to enrollment; antibiotic: time from onset to antibiotic use; SBP: systolic blood pressure on admission; RR: respiratory rate on admission; P: pulse rate on admission; SAP: stroke-associated pneumonia; inpatient: time from onset to hospital; T: temperature on admission; DBP: diastolic blood pressure on admission; CHD: coronary heart disease. **(E)** LEfSe analysis showed significant metabolic pathways among ICH and control groups (LDA scores (log10) > 2.5). (**F**) Heatmap of Spearman’s rank correlation coefﬁcient among significant metabolic pathways and significant microbiota (LDA scores (log10) > 4).

*: P < 0.05, **: P < 0.01.


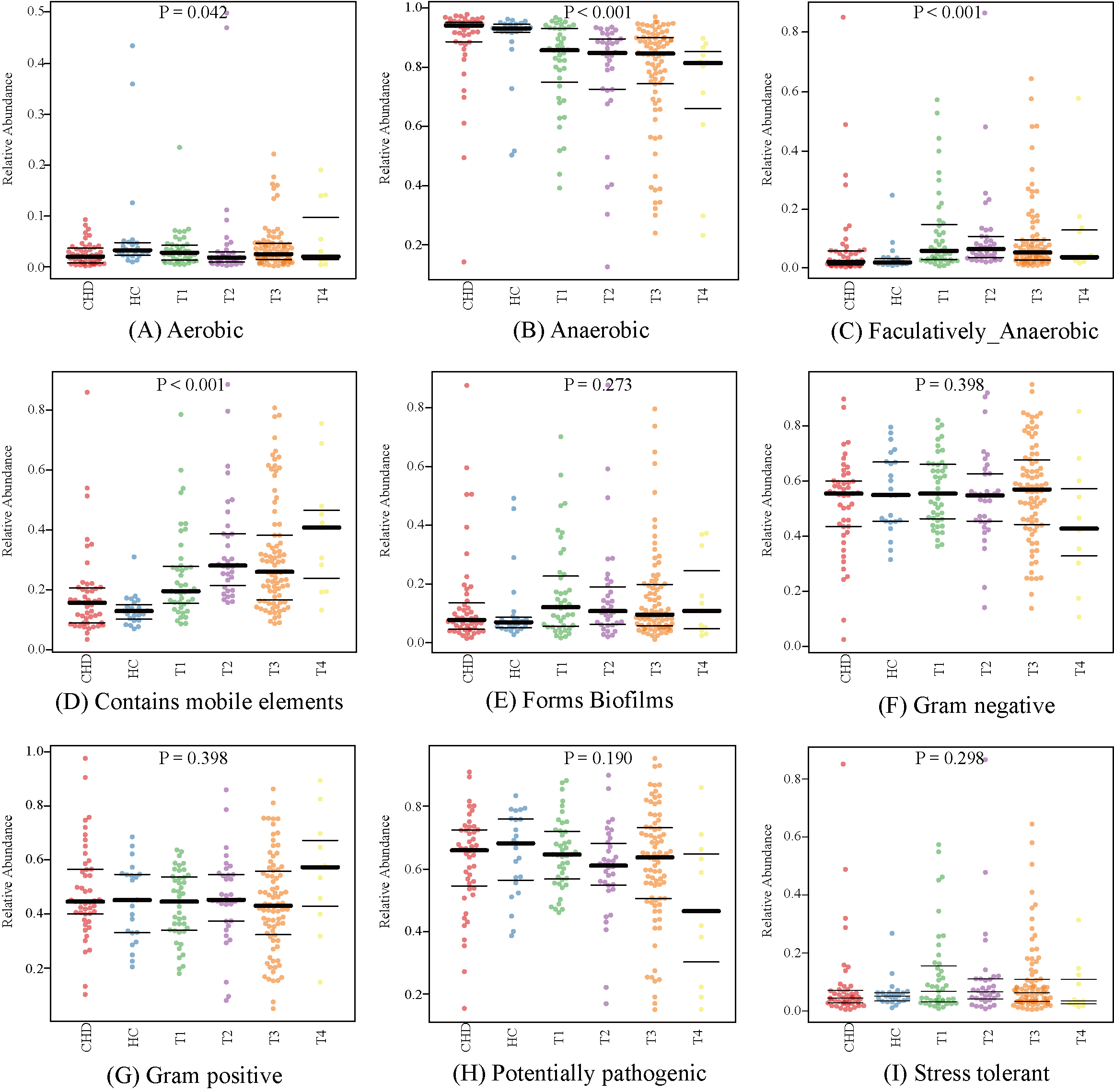


**Figure S3**. BugBase predicted microbial community phenotypes and the corresponding bacterial contributions. **(A–C)** Oxygen utilization. **(D)** Mobile genetic element content. **(E)** Biofilm formation. **(F–G)** Gram bacterial classification. **(H)** Pathogenic risk. **(I)** Oxidative stress tolerance. Statistical significance was determined by Kruskal-Wallis test.


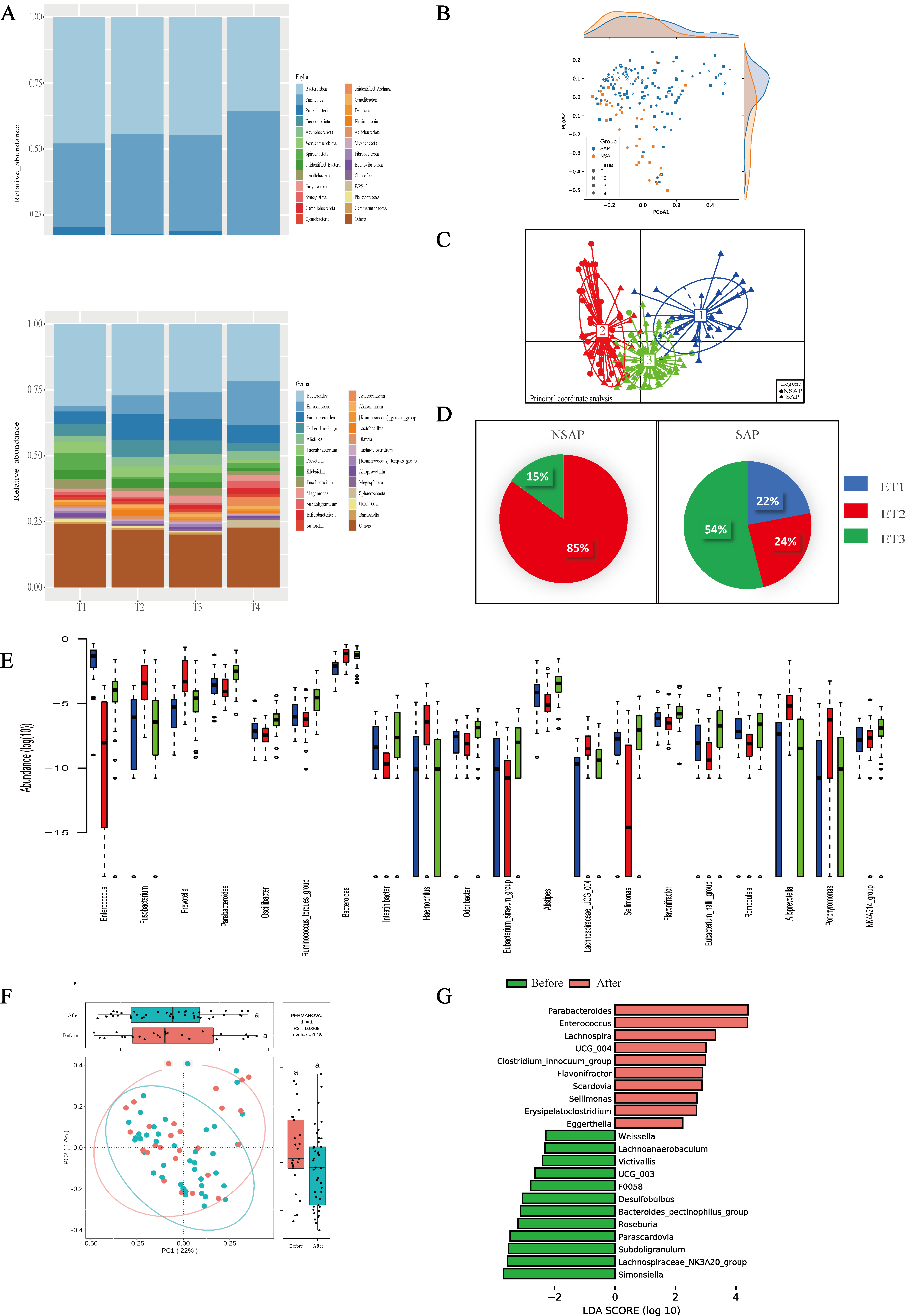


**Figure S4**. Comparison of the microbial communities of the SAP and non-SAP groups. **(A)** Gut microbial composition of SAP groups at the phylum level and genus level. **(B)** PCoA plot with the Bray-Curtis dissimilarities between the SAP (n = 131) and non-SAP group (n = 39) was shown. **(C)** The fecal samples were clustered into three distinct enterotypes (ETs) using the Jensen-Shannon distance metric based on the relative abundances of bacteria at the genus level. Different colors represent enterotypes classified by the partitioning around medoids (PAM) clustering algorithm. ET1 (blue): *Bacteroides*, ET2 (red): *Prevotella*; ET3 (green): *Ruminococcus*. **(D****)** Percentage of ETs in the two groups. **(E)** Top 20 genera in different ETs. ET1 (blue): *Bacteroides*, ET2 (red): *Prevotella*; ET3 (green): *Ruminococcus*. (**F)** PCoA with the Bray-Curtis dissimilarities showing the gut microbiota composition among patients with ICH before and after developing pneumonia. (**G**) LEfSe analysis revealed differences in the gut microbiota between the before and after developing groups in ICH. LDA scores (log10) > 2) and P < 0.05 are shown.


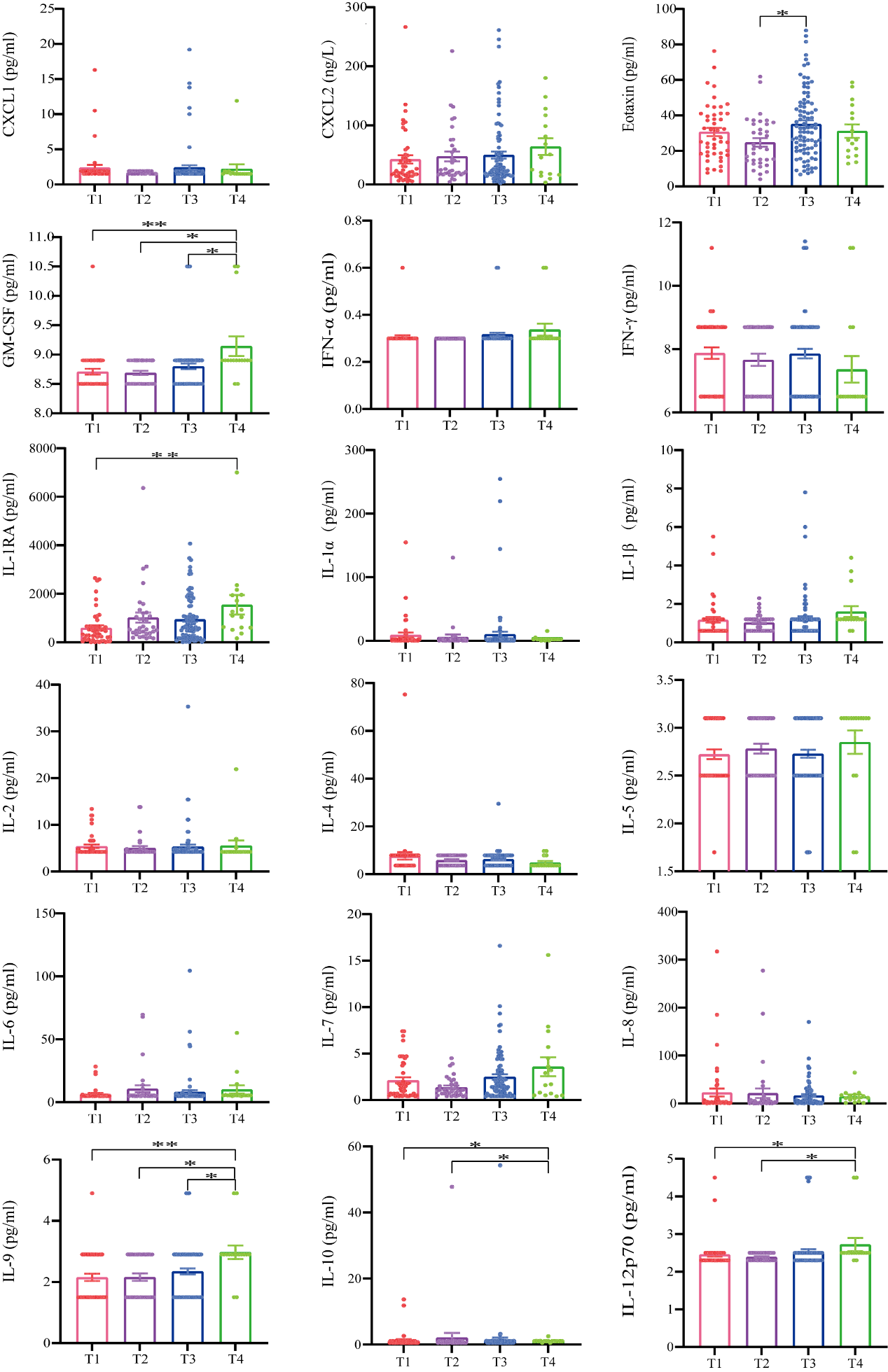


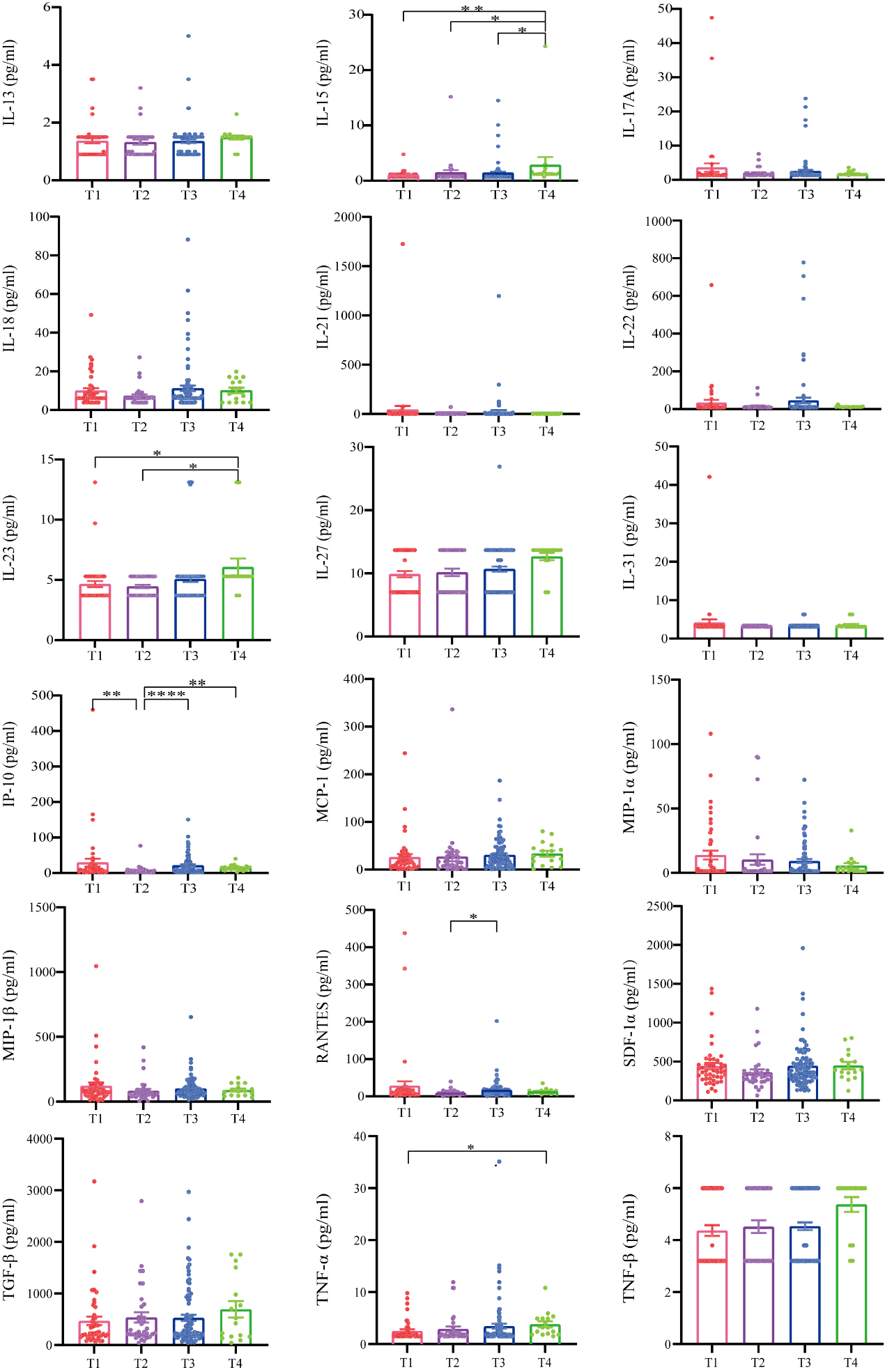


**Figure S5.** Serum concentrations of individual cytokine by time point. There were 12 cytokines changed significantly over time (Kruskal-Wallis Test, Eotaxin: P = 0.036; GM-CSF: P = 0.006; IL-8: P = 0.027; IL-9: P = 0.011; IL-10: P = 0.030; IL-12p70: P = 0.014; IL-15: P = 0.006; IL-23: P = 0.015; IL-1RA: P = 0.003; IP-10: P < 0.0001; RANTES: P = 0.028 and TNF-α: P = 0.012). *: P < 0.05, **: P < 0.01, ****: P < 0.0001.


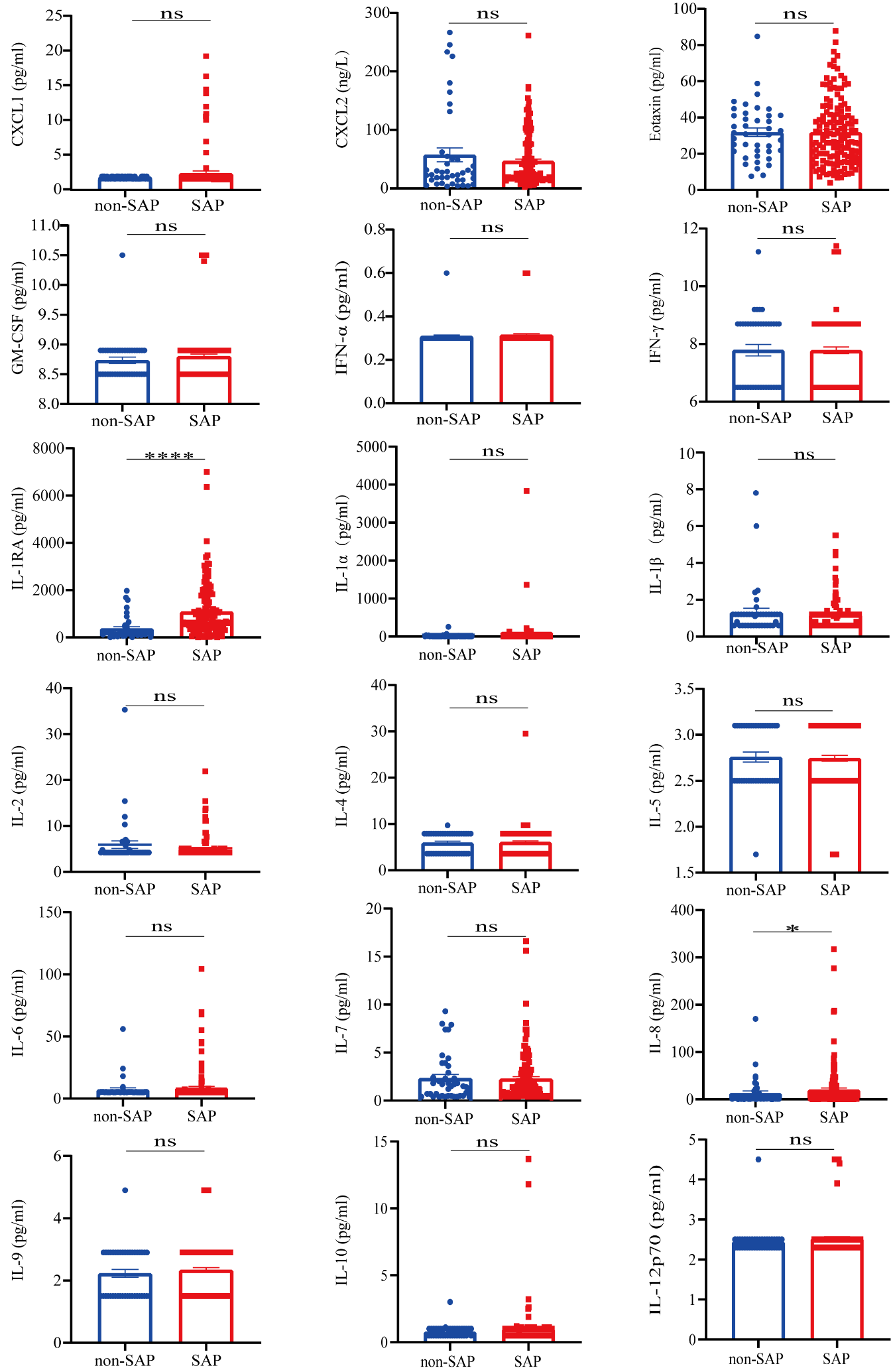


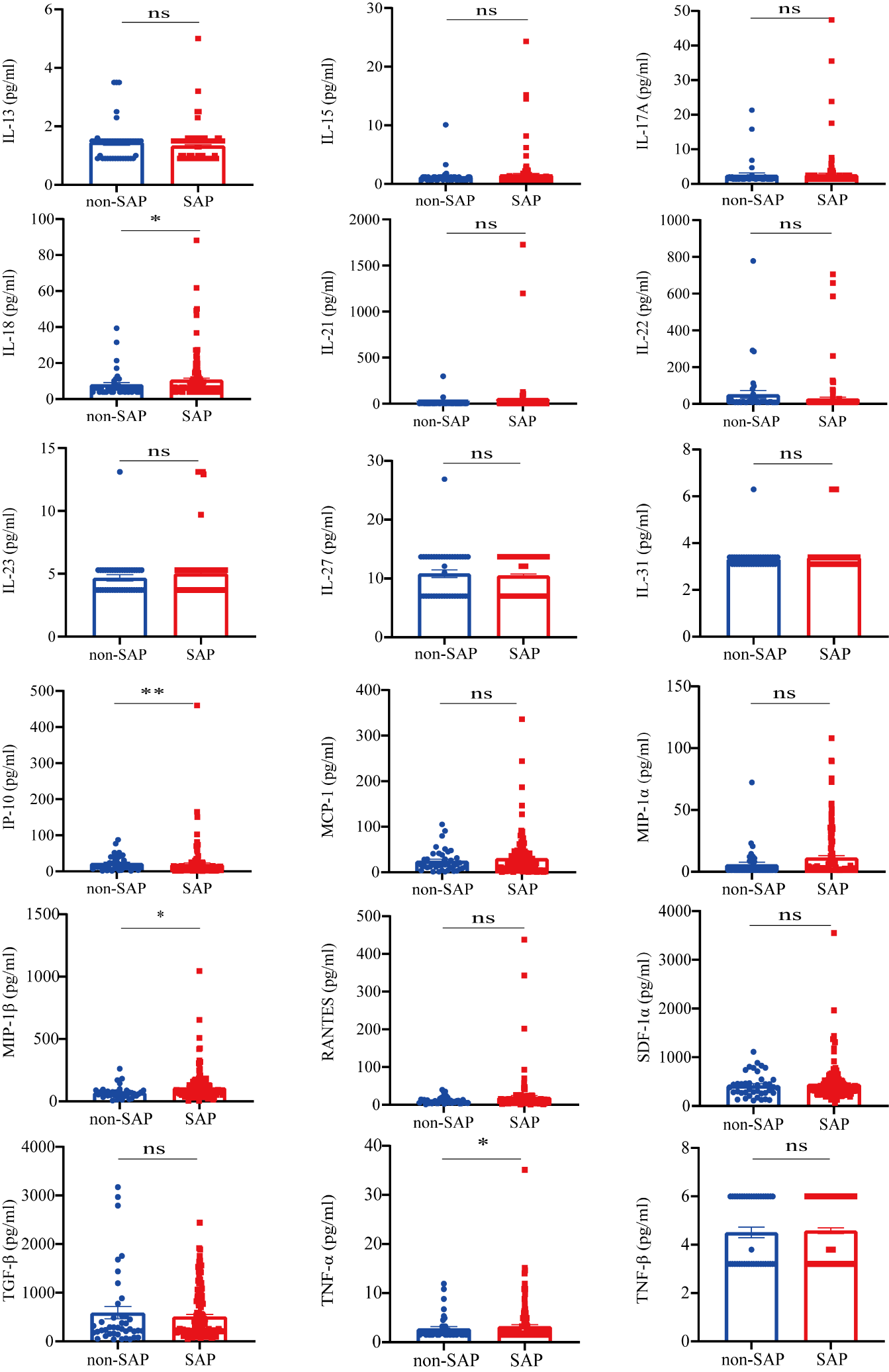


**Figure S6**. Serum concentrations of individual cytokine in SAP (n=143) and non-SAP (n=41) groups. Data were analyzed using unpaired 2-tailed Mann-Whitney U tests. There were significant differences in IP-10 (P = 0.006), IL-8 (P = 0.014), IL-1RA (P < 0.0001), TNF-α (P = 0.045), MIP-1β (P = 0.013), and IL-18 (P = 0.031) levels between the SAP and non-SAP groups. *: P < 0.05, **: P < 0.01, ****: P < 0.0001.
